# Notch3/Hes5 Induces Vascular Dysfunction in Hypoxia-Induced Pulmonary Hypertension Through ER Stress and Redox-Sensitive Pathways

**DOI:** 10.1161/HYPERTENSIONAHA.122.20449

**Published:** 2023-05-31

**Authors:** Hannah E. Morris, Karla B. Neves, Margaret Nilsen, Augusto C. Montezano, Margaret R. MacLean, Rhian M. Touyz

**Affiliations:** 1 Institute of Cardiovascular and Medical Sciences, University of Glasgow, United Kingdom (H.E.M., K.B.N., A.C.M., R.M.T.).; 2 Strathclyde Institute of Pharmacy and Biomedical Sciences, University of Strathclyde, United Kingdom (M.N., M.R.M.).; 3 Research Institute of McGill University Health Centre, McGill University, Canada (R.M.T.).

**Keywords:** calcium signaling, endoplasmic reticulum, hypoxia, pulmonary hypertension, reactive oxygen species

## Abstract

Notch3 (neurogenic locus notch homolog protein 3) is implicated in vascular diseases, including pulmonary hypertension (PH)/pulmonary arterial hypertension. However, molecular mechanisms remain elusive. We hypothesized increased Notch3 activation induces oxidative and endoplasmic reticulum (ER) stress and downstream redox signaling, associated with procontractile pulmonary artery state, pulmonary vascular dysfunction, and PH development.

**METHODS:**

Studies were performed in TgNotch3_R169C_ mice (harboring gain-of-function [GOF] Notch3 mutation) exposed to chronic hypoxia to induce PH, and examined by hemodynamics. Molecular and cellular studies were performed in pulmonary artery smooth muscle cells from pulmonary arterial hypertension patients and in mouse lung. Notch3-regulated genes/proteins, ER stress, ROCK (Rho-associated kinase) expression/activity, Ca^2+^ transients and generation of reactive oxygen species, and nitric oxide were measured. Pulmonary vascular reactivity was assessed in the presence of fasudil (ROCK inhibitor) and 4-phenylbutyric acid (ER stress inhibitor).

**RESULTS:**

Hypoxia induced a more severe PH phenotype in TgNotch3_R169C_ mice versus controls. TgNotch3_R169C_ mice exhibited enhanced Notch3 activation and expression of Notch3 targets Hes Family BHLH Transcription Factor 5 (Hes5), with increased vascular contraction and impaired vasorelaxation that improved with fasudil/4-phenylbutyric acid. Notch3 mutation was associated with increased pulmonary vessel Ca^2+^ transients, ROCK activation, ER stress, and increased reactive oxygen species generation, with reduced NO generation and blunted sGC (soluble guanylyl cyclase)/cGMP signaling. These effects were ameliorated by N-acetylcysteine. pulmonary artery smooth muscle cells from patients with pulmonary arterial hypertension recapitulated Notch3/Hes5 signaling, ER stress and redox changes observed in PH mice.

**CONCLUSIONS:**

Notch3 GOF amplifies vascular dysfunction in hypoxic PH. This involves oxidative and ER stress, and ROCK. We highlight a novel role for Notch3/Hes5-redox signaling and important interplay between ER and oxidative stress in PH.

**NOVELTY AND RELEVANCE**

**What Is New?**

R169C Notch3 (neurogenic locus notch homolog protein 3) mutation induces gain-of-function Notch3-Hes Family BHLH Transcription Factor 5 (Hes5) signaling in the lungs and pulmonary vascular dysfunction.
Notch3-induced dysfunction associates with endoplasmic reticulum/oxidative stress and ROCK (Rho-associated kinase), predisposing to hypoxic pulmonary hypertension (PH).
Elevated endoplasmic reticulum stress/reactive oxygen species associates with increased Notch3 signaling in human pulmonary arterial hypertension pulmonary artery smooth muscle cells.

**What Is Relevant?**

Notch3 contributes to PH by promoting cell proliferation and vascular remodeling. Our data also implicate Notch3 in dysfunctional hypercontractility and vasorelaxation in PH.
These findings connect Notch3 to downstream pathways important in PH and vascular dysfunction, including ROCK, endoplasmic reticulum stress, and reactive oxygen species.

**Clinical/Pathophysiological Implications?**

PH/pulmonary arterial hypertension is debilitating with limited treatments and poor prognosis. Dampening of Notch3 signaling or downstream pathways highlighted in this study may constitute useful future therapeutic targets for PH.

The Notch (neurogenic locus notch homolog protein) family of 4 transmembrane receptors play a key role in mediating cell-cell communication, regulating diverse cell functions including differentiation, maturation, proliferation, and apoptosis.^1^ Notch3 (neurogenic locus notch homolog protein 3) is expressed in vascular smooth muscle cells, regulating proliferation and contraction essential for VSMC function.^2^ As such, aberrant Notch3 signaling is implicated in diseases characterized by vascular remodeling, including cerebral autosomal dominant arteriopathy with subcortical infarctions and leukoencephalopathy (CADASIL) and pulmonary hypertension (PH).^3,4^

Notch3 mutations underlie CADASIL, a cerebral arteriopathy characterized by cerebrovascular dysfunction, stroke and premature vascular dementia.^5^ Notch3 mutations cause CADASIL, likely through gain-of-function (GOF) effects over a loss-of-function mechanism.^6^ The TgNotch3_R169C_ mouse, a transgenic model of GOF Notch3 mutation has been used to study the pathophysiology of Notch3 in CADASIL and other conditions.^7,8^ Here, we studied this model in PH.

PH is defined as mean pulmonary arterial pressure of ≥20 mm Hg, whereas pulmonary arterial hypertension (PAH) describes the progressive and irreversible remodeling disease. PAH is characterized by increased resistance and pathological remodeling in small pulmonary arteries, mediated by vasoconstriction and a proproliferative VSMC phenotype shift. Notch3 is implicated in both processes; however, previous work focuses on Notch3-mediated proliferation in PH.^4^ In human PAH, Notch3 expression correlates positively with vascular resistance.^9^ Notch3 mutations described in PAH are also hypothesized to contribute to pulmonary artery smooth muscle cell (PASMC) proliferation and vasoconstriction.^10,11^ Additionally, hypoxia upregulates Notch3 signaling, further implicating involvement in PAH.^12^ Hypoxia is commonly used to induce experimental PH and increased Notch3 expression and signaling appear necessary for hypoxic PH development in various models, with Notch3 inhibition proving successful as an intervention.^4,9^ Transgenic mice overexpressing the GOF R169C Notch3 mutation (TgNotch3_R169C_) therefore provide a useful model for examining Notch3-mediated downstream signaling in the lung.

Mutant Notch3 can aggregate in and damage the endoplasmic reticulum (ER),^13^ and contribute to peripheral CADASIL vascular dysfunction.^8,14^ ER stress is also implicated in experimental PH^15^ and human Notch3 mutations described in PAH patients also indicate a role for ER chaperones.^16^ ROCK (Rho-associated kinase) is well documented in the pathogenesis of PH, through its effects on vasoconstriction and remodeling^17^ and implicated in the TgNotch3_R169C_ CADASIL phenotype.^8^ Moreover, redox-sensitive RhoA/ROCK acts downstream of Notch3 in myogenic tone regulation.^18^ There is significant interplay between reactive oxygen species (ROS)/reactive nitrogen species and ER stress,^19^ and both processes are implicated in PH models. Additionally, chronic hypoxia contributes to vascular dysfunction alongside these pathways.^20,21^

Despite this known role for Notch3 in pulmonary vascular dysfunction, there is a paucity of information on downstream PASMC signaling, particularly with respect to vascular contraction and dilation. We hypothesized that Notch3 activity is associated with exaggerated pulmonary vascular dysfunction in hypoxia-induced PH and that this involves oxidative and ER stress, activation of redox-sensitive pathways and altered function in VSMCs. We studied TgNotch3_R169C_ mice, which harbor a GOF Notch3 mutation, and tested human relevance in pulmonary artery VSMCs from patients with PAH.

## METHODS

### Data Availability

See Supplemental Material for detailed methods. Data supporting the findings of this study are available from the corresponding author upon reasonable request.

### Notch3 Mutant Mouse Model and Animal Studies

The transgenic TgNotch3_WT_ and TgNotch3_R169C_ mouse lines have been characterized previously and TgNotch3_R169C_ mice exhibit CADASIL features by 6 months.^7,22^ Females were excluded for potential transgene mosaicism.^23^

Mice were challenged with 14 days hypobaric hypoxia (550 mbar/≈10% O_2_) to induce moderate PH, previously described.^24^ PH was assessed by in vivo hemodynamic measurements, right ventricular hypertrophy, and histopathologic and immunohistochemistry analysis of lung sections, as described^24^ and in the Supplemental Material.

### Pulmonary Vascular Functional Studies by Wire Myography

Intralobar pulmonary arteries (≈350 μm internal diameter) were isolated from normoxic and hypoxic TgNotch3_WT_ and TgNotch3_R169C_ mice as described^25^ and Supplemental Material. Cumulative concentration responses curves were constructed in response to 5-hydroxytryptamine, endothelin 1, acetylcholine, and sodium nitroprusside (SNP), with and without fasudil and 4-phenylbutyric acid (4-PBA) preincubation.

### PASMC Isolation and Culture

Mouse PASMCs were extracted from TgNotch3 pulmonary arteries and cultured with an adapted method described before.^26^ Human PASMCs were isolated from distal pulmonary arteries (≤1 mm) from patients with PAH and controls (non-PAH individuals) as previously described^27^ (Professor Nicholas Morrell, Cambridge, United Kingdom).

### Measurement of Intracellular Ca^2+^ Transients in Mouse PASMC

Intracellular free Ca^2+^ concentration (intracellular calcium levels ([Ca^2+^]i) was measured in TgNotch3 PASMCs using the fluorescent Ca^2+^ indicator, Cal‐520 am as previously described,^28^ after stimulation with 5-5-hydroxytryptamine or endothelin 1 (in some cases with 4-PBA/N-acetylcysteine pretreatment).

### Quantitative Real-Time Polymerase Chain Reaction

Gene expression analysis was by quantitative real-time polymerase chain reaction with SYBR green reagents, calculated by 2^-ΔΔ^^Ct^ fold change. Primers in Table S1.

### Immunoblotting

Immunoblotting was performed for proteins involved in Notch3-Hes Family BHLH Transcription Factor 5 (Hes5), ROCK, NO/cGMP pathway, ER stress, and redox signaling. Antibodies are detailed in Table S2.

### ROS Measurements

ROS was assessed in lung tissue and PASMCs. Lucigenin-enhanced chemiluminescence was used for ROS generation as described previously.^19^ H_2_O_2_ was assessed by Amplex Red assay, ONOO^-^ by ELISA for 3-nitrotyrosine modified proteins, NO by Total NOx Assay Kit, and lipid peroxidation by malondialdehyde assay, all to manufacturer’s instructions. Protein sulfenylation was by affinity capture immunoblot.

### Statistical Analysis

Data are represented as mean±SEM. Analysis by Student *t* test or 1-way ANOVA with Bonferroni post hoc test as appropriate, or by nonlinear regression for myography. *P<0.05 was considered significant.

## RESULTS

### R169C Mutation Is Associated With Increased Pulmonary Notch3 Signaling

We confirmed overexpression of rat Notch3 transgene in TgNotch3_WT_ and TgNotch3_R169C_ relative to nontransgenic FVB littermates (Figure S1A). To assess GOF, we examined elements of the canonical Notch3 pathway, including the full-length Notch3 receptor protein and a transmembrane intracellular fragment produced by receptor cleavage. We confirm increased Notch3 signaling in TgNotch3_R169C_ lung, in line with work in other peripheral vessels from this model^8^ (Figure S1B). Similarly, downstream Notch3 target Hes5 was shown to be significantly increased in lung from TgNotch3_R169C_ mice compared to controls (Figure S1C and S1D). Notch3 signaling also induces its own transcription, and expression of murine Notch3 was found to be increased in TgNotch3_R169C_ mice (Figure S1E and S1F). Our results demonstrate that, despite transgene overexpression in both strains, only in TgNotch3_R169C_ lung was this associated with elevated Notch3 targets, indicating R169C mutation has a GOF effect on Notch3 signaling.

### Hypoxia Recapitulates Abberant Notch3-Hes5 Signaling and Vascular Reactivity in TgNotch3_WT_ and Amplifies PH Phenotype in TgNotch3_R169C_

**Figure 1.**
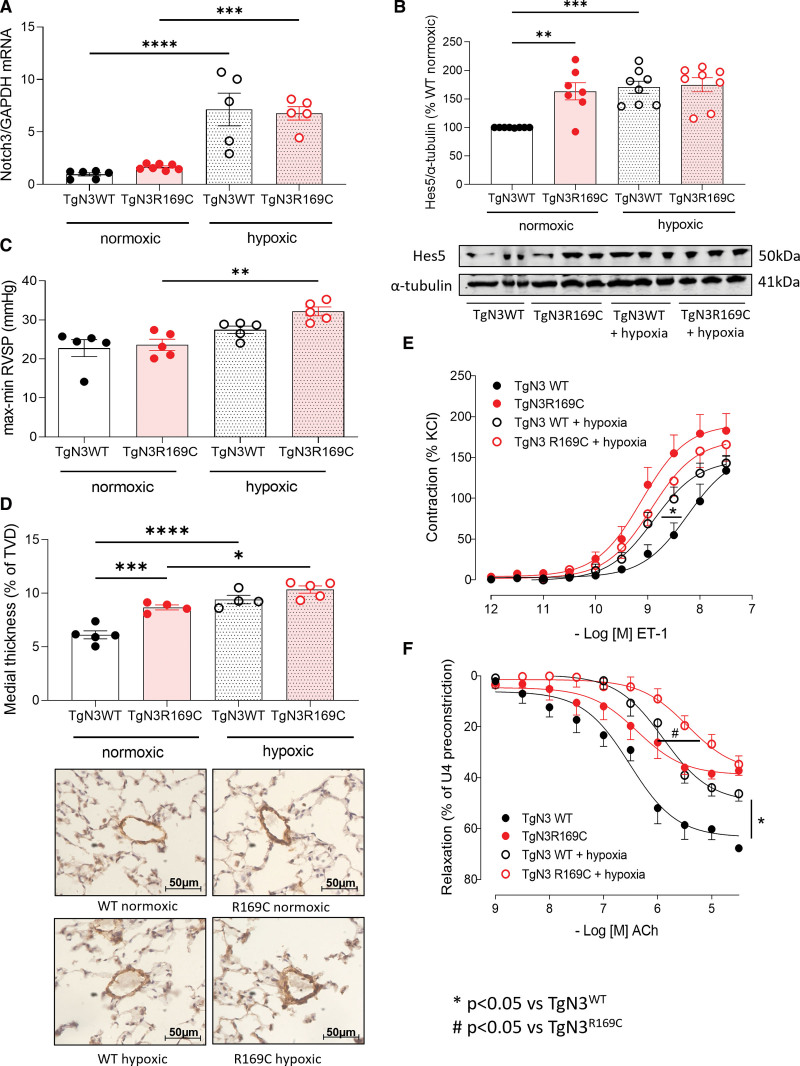
**Hypoxia recapitulates increased gain-of-function (GOF) Notch3 (neurogenic locus notch homolog protein 3)-Hes Family BHLH Transcription Factor 5 (Hes5) axis signaling and impaired vascular function in TgNotch3_WT_ mice and induces a more pronounced hypoxic pulmonary hypertension (PH) phenotype in TgNotch3_R169C_ mice.** TgNotch3 mice were exposed to 14 days of chronic hypobaric hypoxia (10%). Notch3 signaling was assessed in the lung. **A**, Notch3 mRNA expression by real-time quantitative polymerase chain reaction normalized to GAPDH (n=5–7; 1-way ANOVA with Bonferroni post-test). **B**, Notch3 target Hes5 protein expression by immunoblot normalized to α-tubulin. **Upper**, Quantification of Hes5. **Lower**, Representative Hes5 immunoblot. n=8; 1-way ANOVA with Bonferroni post-test. Hemodynamics were measured in vivo via right-heart catheterization. **C**, Right ventricular systolic pressure (RVSP) (n=5; 1-way ANOVA with Bonferroni post-test). **D**, Small pulmonary artery medial layer thickness by α-SMA (α-smooth muscle actin) immunohistochemistry. **Upper**, Semiquantitative medial layer thickness analysis (% total vessel diameter [TVD])±hypoxia. **Lower**, Representative images of increasing medial layer thickness (n=4–5 per group, average of 6 vessels in triplicate per animal). **E** and **F**, Pulmonary artery wire myography in normoxic and hypoxic TgNotch3 mice. Cumulative concentration-response curves of contraction to (**E**) endothelin 1 (ET-1), and relaxation of U46619 preconstriction to (**F**) acetylcholine (Ach; n=3–9; nonlinear regression). Results are mean±SEM. *P<0.05 vs TgNotch3_WT_, #*P*<0.05 vs TgNotch3_R169C_. WT indicates wild type.

Following hypoxia, Notch3 protein expression increased in both strains (Figure 1A; Figure S2A). Gene expression for endogenous murine Notch3, Hes5, and HeyL, downstream targets upregulated by Notch3 signaling, was also higher (Figure 1B; Figure S2B and S2C). Right ventricular systolic pressure was significantly increased in TgNotch3_R169C_ animals after 2 weeks of hypoxia, while right ventricular systolic pressure in TgNotch3_WT_ animals was not significantly increased (Figure 1C). Additionally, hypertrophic remodeling (right ventricular/[left ventricular+S]) was significantly increased in the hypoxic TgNotch3_R169C_ but not hypoxic TgNotch3_WT_ (Figure S2D). Distal pulmonary artery (<80 μm) remodeling marginally increased following hypoxia, without difference between strains (Figure S2E). Medial layer thickness, however, was already increased in TgNotch3_R169C_ pulmonary arteries compared to TgNotch3_WT_ in normoxia. Hypoxia increased medial layer thickness in both strains, with further elevation in TgNotch3_R169C_ (Figure 1D). Hypoxia increased TgNotch3_R169C_ right ventricular contractility but not relaxation (Figure S2F and S2G).

Vascular reactivity by pulmonary artery wire myography demonstrated increased contractile responses to endothelin 1 (Figures 1E) and 5-hydroxytryptamine (Figure S3A), impaired relaxation to SNP (Figure S3B), and impaired endothelial-dependent relaxation to acetylcholine (Figure 1F) in R169C mutant versus wild-type mice at normoxic baseline. In TgNotch3_WT_ vessels, hypoxia then increased contractile responses, whereas TgNotch3_R169C_ vessel responses remained similar to elevated normoxic responses (Figure 1E; Figure S3A). Both strains exhibited diminished relaxation following hypoxia, with further blunting of vasorelaxation in TgNotch3_R169C_ arteries (Figure 1F; Figure S3B).

### Baseline Hypercontractility of Pulmonary Arteries From Notch3 GOF Transgenic Mice Is Mediated by Calcium-Dependent and -Independent Signaling

**Figure 2.**
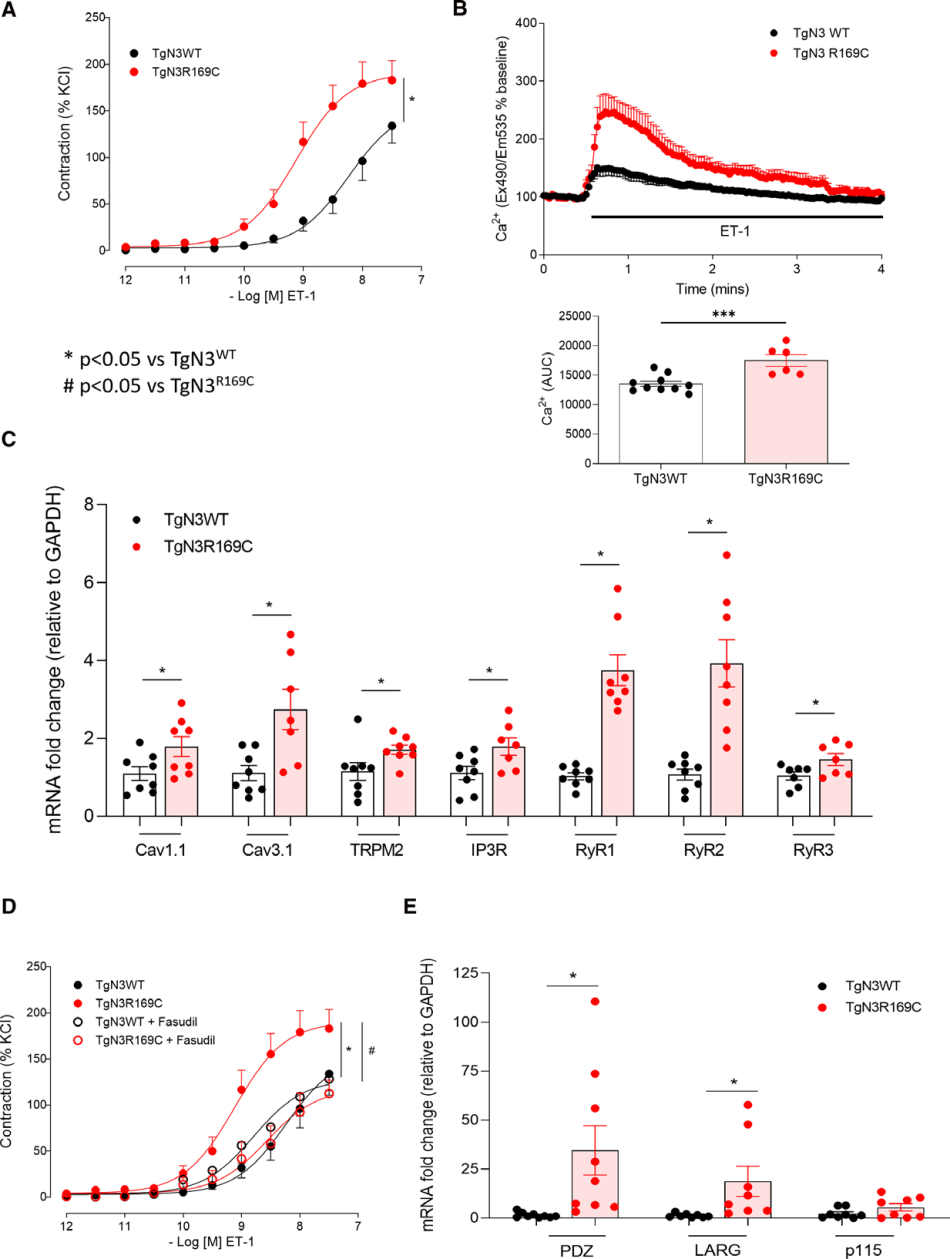
**Baseline pulmonary vascular contraction is enhanced in TgNotch3_R169C_ mice alongside altered calcium-dependent and -independent contractile mechanisms.** Vascular reactivity was assessed in TgNotch3 pulmonary arteries by wire myography±fasudil (1 μmol/L), inhibitor of ROCK (Rho-associated kinase). **A**, Cumulative concentration-response curve to endothelin-1 (ET-1; n=7–9; nonlinear regression). **B**, Intracellular Ca^2+^ transients to ET-1 in TgNotch3 pulmonary artery smooth muscle cells (PASMCs) by live-cell fluorescence. **Upper**, Pulmonary artery smooth muscle cell (PASMC) intracellular calcium levels ([Ca^2+^]i) responses (ET-1, 100 nmol/L). **Lower**, [Ca^2+^]i expressed as area under the curve (AUC; n=6; 1-way ANOVA with Bonferroni post-test). **C**, L-type calcium channel, subunit α1S (Cav1.1), T-type calcium channel, subunit α1G (Cav3.1), IP3R (inositol 1,4,5-trisphosphate receptor), TRPM2 (voltage-dependent Transient receptor potential cation channel, subfamily M, member 2), RyR1 (ryanodine receptor 1), RyR2, and RyR3 Ca^2+^ channel gene expression by real-time quantitative polymerase chain reaction, normalized to GAPDH, in TgNotch3 lung (n=7–8; unpaired *t* test). **D**, Cumulative concentration-response curves to endothelin-1 (ET-1) following fasudil pretreatment (n=7–9; nonlinear regression). **E**, Gene expression for PDZ, LARG, and p115 Rho-GEFs, normalised to GAPDH (n=8–9; unpaired *t* test). Results are mean±SEM. *P<0.05 vs TgNotch3_WT_, #*P*<0.05 vs TgNotch3_R169C_. Larg indicates leukemia-associated RhoGEF; Pdz, Pdz RhoGEF; and wt, wild type.

Contractile mechanisms including intracellular Ca^2+^, ER stress, and ROCK were examined to account for increased Emax and reduced EC_50_ for endothelin 1 and 5-hydroxytryptamine (Figure 2A; Figure S4A through S4C). Ligand-induced Ca^2+^ transients were higher in PASMCs isolated from TgNotch3_R169C_ than TgNotch3_WT_ (Figure 2B; Figure S4D), alongside elevation of several Ca^2+^ channel genes. Plasma membrane channels L-type calcium channel, subunit α1S (Cav1.1), T-type calcium channel, subunit α1G (Cav3.1), and TRPM2 (voltage-dependent Transient receptor potential cation channel, subfamily M, member 2) mRNAs were more abundantly expressed in TgNotch3_R169C_ lung versus TgNotch3_WT_ (Figure 2C). Moreover, ER-associated Ca^2+^ channels, IP3R (inositol 1,4,5-trisphosphate receptor), and RyR (ryanodine receptors) 1, 2, and 3, were elevated in TgNotch3_R169C_ (Figure 2C). KCl-induced contraction, mediated by voltage-gated Ca^2+^ channels, was similar between strains (Figure S4E).

Ca^2+^-independent signaling involving ROCK was also studied. TgNotch3_R169C_ artery hypercontractility was attenuated with pretreatment of fasudil, without significant effect on TgNotch3_WT_ vessels (Figures 2D, Figure S5A). ROCK1/2 expression was unaltered by Notch3 mutation (Figure S5B and S5C). GEFs (guanine nucleotide-exchange factors), which positively regulate RhoGTPase activity, were then assessed. Gene expression for Pdz (Pdz RhoGEF) and Larg (leukemia-associated RhoGEF) was increased in TgNotch3_R169C_ versus TgNotch3_WT_; p115 Rho GEF was unchanged (Figure 2E). Hypoxia upregulated ROCK mRNA expression, particularly in TgNotch3_R169C_ mice (Figure S6A and S6B). Inhibiting ROCK with fasudil also improved hypoxia-induced alterations to pulmonary vascular reactivity in both TgNotch3 strains exposed to hypoxia (Figure S6C and S6D).

### Notch3 GOF Induces ER and Oxidative Stress

**Figure 3.**
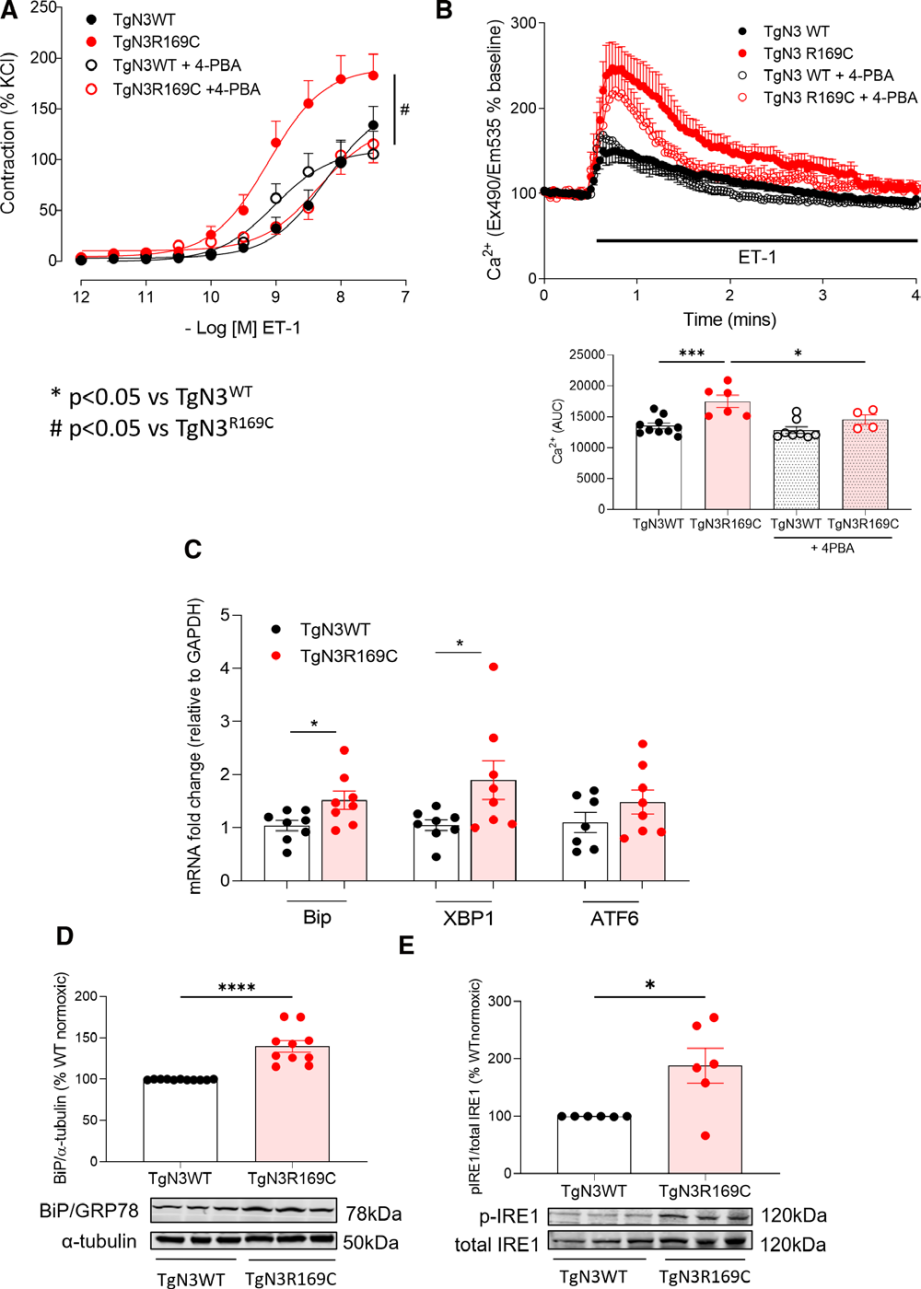
**Endoplasmic reticulum (ER) stress in TgNotch3_R169C_ mice contributes to altered pulmonary vascular reactivity.** Vascular contraction to endothelin 1 (ET-1; **A**) was assessed by TgNotch3 pulmonary artery wire myography±ER stress inhibitor 4-phenylbutyric acid (4-PBA; 1 mmol/L). n=7–9; nonlinear regression. **B**, Intracellular Ca^2+^ transients to ET-1 in TgNotch3 pulmonary artery smooth muscle cells (PASMCs) by live-cell fluorescence±24-hour 4-PBA (1 mmol/L). **Upper**, Representative PASMC intracellular calcium levels ([Ca^2+^]i) responses to ET-1 (100 nmol/L) +24-hour 4-PBA. **Lower**, [Ca^2+^]i expressed as area under the curve (AUC; n=6; 1-way ANOVA with Bonferroni post-test). ER stress markers were assessed by real-time quantitative polymerase chain reaction and immunoblotting in TgNotch3 lung. **C**, Gene expression for BiP (binding immunoglobulin protein), XBP1 (X-box binding protein 1), and activating transcription factor 6 (ATF6) normalized to GAPDH (n=8; unpaired *t* test). **D**, **upper**, Quantification of ER chaperone BiP protein normalised to α-tubulin (n=11; unpaired *t* test)**. Lower**, representative BiP immunoblot. **E**, **upper**, quantification of ER sensor IRE1 (inositol-requiring enzyme 1) phosphorylation (p-IRE1) normalised to total IRE1 (n=6; unpaired *t* test). **Lower**, Representative p-IRE1 immunoblot. Results are mean±SEM. *P<0.05 vs TgNotch3_WT_, #*P*<0.05 vs TgNotch3_R169C_. WT indicates wild type.

In pulmonary vascular reactivity studies, ER stress inhibitor 4-PBA normalized TgNotch3_R169C_ hypercontractility, without effect in TgNotch3_WT_ and attenuated increased ligand-induced Ca^2+^ transients in isolated TgNotch3_R169C_ PASMCs to TgNotch3_WT_ level (Figure 3A and 3B; Figure S7A and S7B). ER stress–associated genes for BiP (binding immunoglobulin protein) and XBP1 (X-box binding protein 1) mRNAs were upregulated in TgNotch3_R169C_ lung (Figure 3C). Immunoblotting confirmed increased BiP expression and enhanced phosphorylation of ER sensor IRE1 (inositol-requiring enzyme 1) in TgNotch3_R169C_ at baseline (Figure 3D and 3E). Hypoxia recapitulated elevated pulmonary BiP expression in TgNotch3_WT_ mice (Figure S8A). Hypoxia-induced hypercontractility in both strains was reduced by 4-PBA (Figure S8B and S8C).

**Figure 4.**
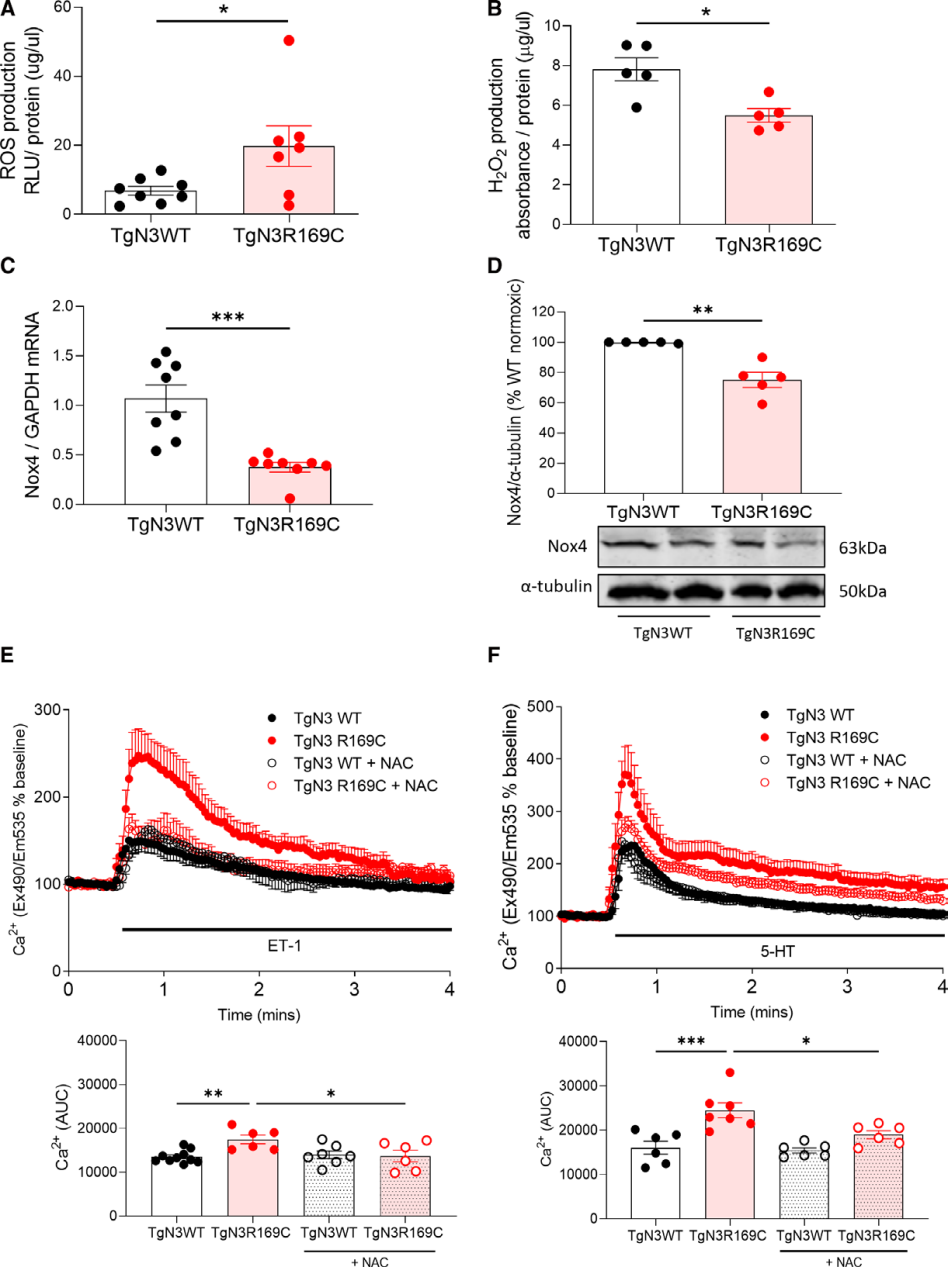
**TgNotch3_R169C_ gain-of-function mutation is associated with altered redox signaling and reactive oxygen species (ROS) levels.** ROS production was measured in TgNotch3 lung by lucigenin-enhanced chemiluminescence and Amplex red assay, lipid peroxidation was assessed TBARS assay normalized by total protein. **A**, ROS production by lucigenin (n=7–8; unpaired *t* test). **B**, H_2_O_2_ levels by Amplex red (n=5; unpaired *t* test). Nox4 (NAPDH oxidase 4) expression in TgNotch3 lung was assessed by (**C**) real-time quantitative PCR normalized to GAPDH (n=5–8; unpaired *t* test) and (**D**) immunoblot normalized to α-tubulin. **D**, **Upper**, Quantification of Nox4 protein expression. **Lower**, Representative Nox4 immunoblot (n=5; unpaired *t* test). TgNotch3 pulmonary artery smooth muscle cell (PASMC) intracellular Ca^2+^ transients to (**E**) endothelin 1 (ET-1) and (**F**) 5-hydroxytryptamine (5-HT) were measured by live-cell fluorescence ±24-hour antioxidant N-acetylcysteine (NAC; 10 µmol/L). **Upper**, PASMC intracellular calcium levels ([Ca^2+^]i) responses to ET-1 (100 nmol/L) or 5-HT (1 μmol/L) +24-hour NAC. **Lower**, [Ca^2+^]i calculated as area under the curve (n=6; 1-way ANOVA with Bonferroni post-test). Results are mean±SEM. *P<0.05. AUC indicates area under the curve; RLU, relative light units; and WT, wild type.

ER stress and ROS are interlinked. Therefore redox-sensitive processes in TgNotch3_R169C_ mice were examined. Levels of O_2_^-^ (Figure 4A) and lipid peroxidation (Figure S9A), were increased in TgNotch3_R169C_. Conversely, TgNotch3_R169C_ pulmonary H_2_O_2_ production was decreased (Figure 4B) alongside reduced expression of H_2_O_2_-producing Nox4 (NAPDH oxidase 4; Figure 4C and 4D). Expression of SOD (superoxide dismutase) isoforms, which convert O_2_^-^ to H_2_O_2_, did not differ between strains (Figure S9B through S9E). In isolated TgNotch3_R169C_ PASMCs, increased ligand-induced [Ca^2+^]i transients significantly reduced with ROS scavenger N-acetylcysteine (Figure 4E and 4F).

### Impaired Vasorelaxation of Pulmonary Arteries in Notch3-Mutant Mice Involves ROS

**Figure 5.**
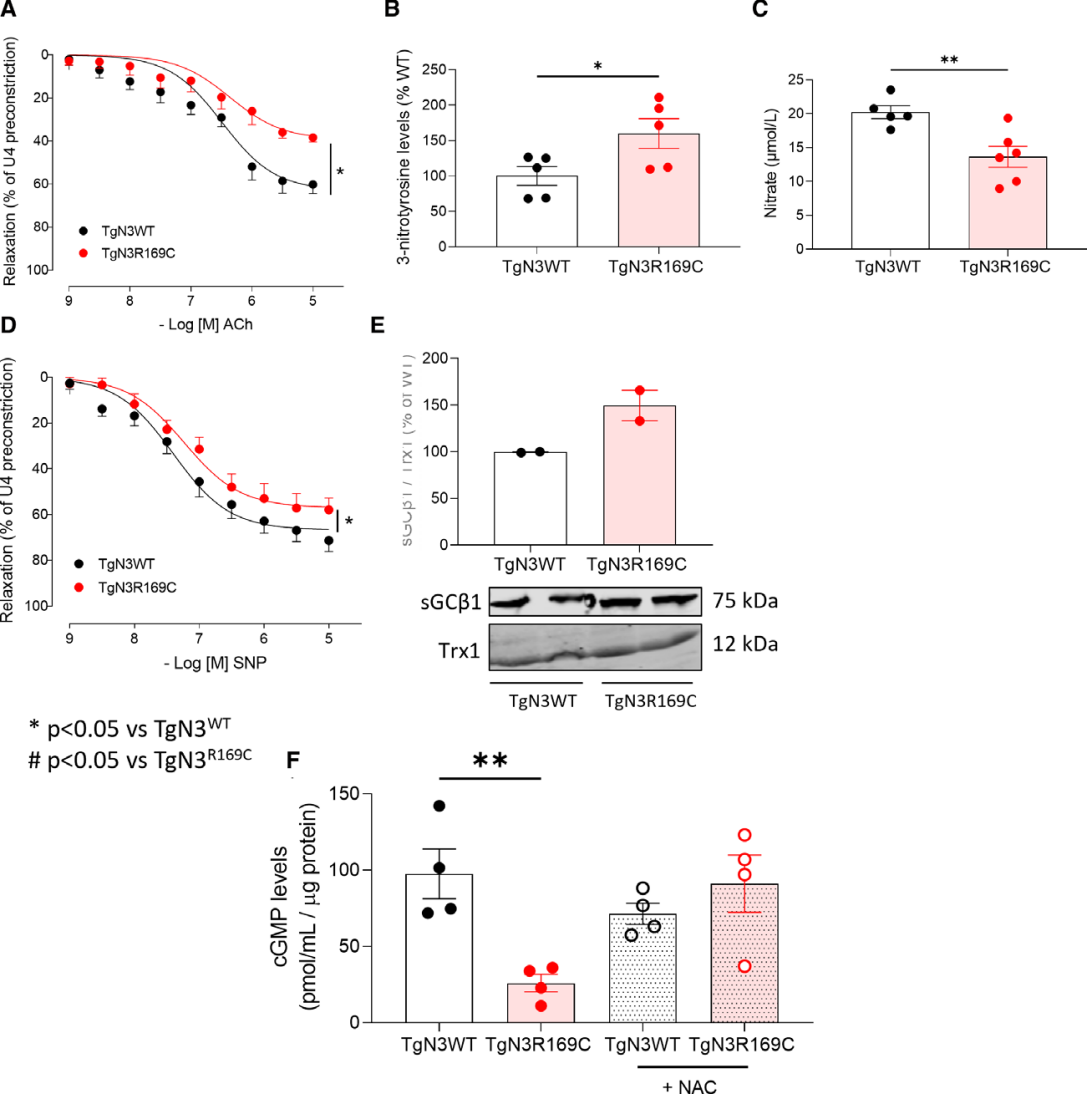
**Impaired endothelial-dependent and -independent vasorelaxation in TgNotch3_R169C_ arteries involves altered NO/cGMP signaling.** Vascular reactivity was assessed in TgNotch3 pulmonary arteries by wire myography, responses expressed as percentage relaxation of preconstriction. **A**, Endothelium-dependent acetylcholine (ACh) vasorelaxation (n=7–9; nonlinear regression). **B**, Levels of 3-nitrotyrosine modified proteins in TgNotch3 lung, normalized to total protein (n=5; unpaired *t* test). **C**, NO levels by total nitrite/nitrate in TgNotch3 lung. **D**, Endothelium-independent sodium nitroprusside vasorelaxation in TgNotch3 pulmonary arteries (n=9–10; nonlinear regression). **E**, Reversible sGCβ1 (soluble guanylyl cyclase β1) oxidation by affinity capture of sulfenylated proteins in whole lung from TgNotch3_WT_ and TgNotch3_R169C_ mice (pool of 3 animals per sample). **F**, cGMP ELISA in TgNotch3 pulmonary artery smooth muscle cells±antioxidant N-acetylcysteine (NAC; 10 µM). Results are mean±SEM. *P<0.05 vs TgNotch3_WT_, **P<0.01 vs TgNotch3WT.

We then investigated Notch3 GOF mutation effects on vascular relaxation in TgNotch3 mice by pulmonary artery wire myography. In Notch3-mutant mice arteries, acetylcholine-induced endothelial-dependent relaxation was significantly impaired compared with wildtype (Figure 5A), ameliorating with fasudil or 4-PBA (Figure S10A and S10B). Notch3 mutation did not affect expression of eNOS (endothelial nitric oxide synthase; Figure S10C), or phosphorylation of eNOS at its activator (Ser_1177_) or inhibitory site (Thr_495_; Figure S10D and S10E). Nitrosylated tyrosine residues, indicating peroxynitrite (ONOO^−^) modification, were significantly more abundant in Notch3-mutant lung (Figure 5B), alongside lower NO (NOx assay; Figure 5C). Fasudil and 4-PBA had similar effects to restore further impaired acetylcholine-mediated relaxation in arteries from hypoxic TgNotch3 mice, in the context of increased ROS and reduced NO (but without alteration to H_2_O_2_) in TgNotch3_WT_ mice (Figure S11A through S11D).

TgNotch3_R169C_ arteries also exhibited reduced endothelial-independent SNP vasorelaxation compared to TgNotch3_WT_ (Figure 5D); again, this improved with fasudil or 4-PBA (Figure S12A and S12B). Downstream NO target sGC (soluble guanylyl cyclase) expression was unaffected by Notch3 mutation (Figure S12C through S12E); however, a small increase in reversible oxidation of sGCβ1 was observed (Figure 5E). Vasodilatory cGMP levels were concordantly lower in isolated TgNotch3_R169C_ versus TgNotch3_WT_ PASMCs, improving following N-acetylcysteine incubation (Figure 5F). Again, fasudil and 4-PBA also improved SNP vasorelaxation in both strains following further impairment by hypoxia (Figure S12F and S12G).

### PASMCs From Patients With PAH Reflect Aberrant Signaling in TgNotch3_R169C_ Mice and Hypoxia

**Figure 6.**
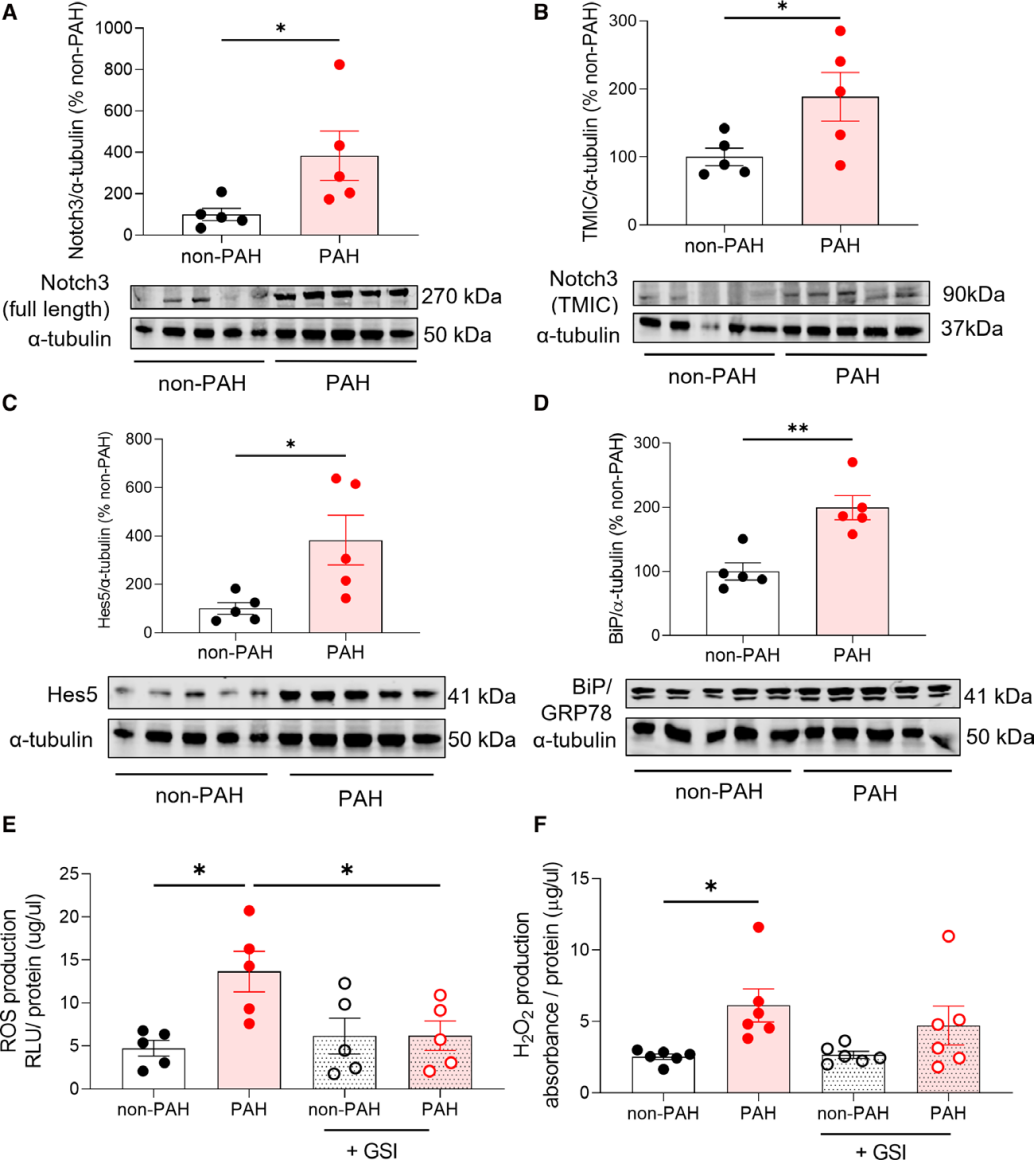
**Upregulation of signaling associated with Notch3 (neurogenic locus notch homolog protein 3)-Hes Family BHLH Transcription Factor 5 (Hes5), endoplasmic reticulum (ER) stress, and reactive oxygen species (ROS) in pulmonary artery smooth muscle cells (PASMCs) from patients with pulmonary arterial hypertension (PAH).** Protein expression in PAH vs non-PAH PASMCs by immunoblot normalized to α-tubulin, data expressed as % of non-PAH, comparisons by unpaired *t* test (n=5). **A**, **upper**, Quantification of Notch3 protein in PAH vs non-PAH PASMCs. **Lower**, Representative immunoblot of total Notch3. **B**, **upper**, Quantification of Notch3 transmembrane intracellular (TMIC) domain. **Lower**, Representative immunoblot of Notch3 TMIC. **C**, **Upper**, Quantification of Notch3 target Hes5. **Lower**, Representative Hes5 immunoblot. **D**, **Upper**, Quantification of ER stress chaperone BiP (binding immunoglobulin protein). **Lower**, Representative BiP immunoblot. **E**, NADPH-dependent ROS and (**F**) H_2_O_2_ by lucigenin and Amplex Red assay respectively ±24-hour Notch inhibitor GSI (n=5 per group, 1-way ANOVA with Bonferroni postcorrection). GSI indicates γ-secretase; and RLU, relative light units. Results are mean±SEM. *P<0.05.

To assess the clinical relevance of our studies we examined pathways elucidated in the TgNotch3_R169C_ model in the context of human PAH, using PASMCs isolated from individuals with and without PAH (patient characteristics in Table S3). PAH PASMCs show increased expression of total Notch3 and TMIC fragment (Figure 6A and 6B), indicating increased expression and activation in PAH. Previous studies implicated Hes5 as a key Notch3 target in PAH,^9^ and expression was higher in PAH-derived versus non-PAH cells (Figure 6C). Consistent with R169C mouse findings, key ER stress protein BiP was upregulated in PAH cells (Figure 6D). Elevated NADPH-dependent ROS observed in TgNotch3_R169C_ lungs was also recapitulated in PAH cells, and was abrogated by γ-secretase inhibitor (GSI) treatment to inhibit Notch (Figure 6E). However, unlike in our model, H_2_O_2_ was increased in PAH PASMCs and unaffected by GSI (Figure 6F). Nox4 protein expression was concurrently elevated (Figure S13).

## DISCUSSION

Major findings from our study demonstrate that GOF Notch3 mutation exacerbates development of PH with associated hypercontractility and impaired relaxation in pulmonary arteries. These processes involve oxidative and ER stress, Ca^2+^ and ROCK signaling, and impaired NO/cGMP signaling. Moreover, molecular changes observed in hypoxia-induced PH in TgNotch3_R169C_ mice were recapitulated in PASMCs from PAH patients. Our investigations define novel Notch3-redox-regulated pathways underlying pulmonary vascular dysfunction and highlight ER stress and oxidative stress interplay as potential major drivers of these processes in PH.

To explore the potential role of Notch3 in PH pathophysiology in the intact system, we studied TgNotch3 mice exposed to hypoxia. Hypoxia increased pulmonary Notch3-Hes5 signaling in TgNotch3_WT_ with exaggerated effects in TgNotch3_R169C_ mice. This was associated with worsening PH, as evidenced by increased right ventricular systolic pressure and hypertrophic remodeling. Together with these changes, pulmonary vessels in hypoxic mice exhibited marked hypereactivity and reduced vasorelaxation, responses that were amplified in TgNotch3 mice versus wild type. Unlike previous studies demonstrating severe PH in chronic-hypoxic mice, TgNotch3_R169C_ mice exhibited a mild-moderate phenotype. This may relate to the FVB background strain, previously defined as a low-responder to hypoxia.^29^ It is also possible that a 14-day hypoxia duration was too short to induce robust PH. Nevertheless, the more pronounced phenotype in Notch3-mutant mice was consistent with our hypothesis that hypoxia exaggerates aberrant Notch3 signaling, amplifying the GOF vascular response evidenced by baseline TgNotch3_R169C_ hemodynamic and remodeling changes. This may involve upregulation of α-SMA (α-smooth muscle actin), a Notch3 signaling procontractile target.^30,31^

Hypoxia is a potent inducer of oxidative stress^32^ as observed in our study. Hypoxic alterations to vascular reactivity improved with fasudil and 4-PBA, suggesting a role for ROCK and ER stress in PH vascular dysfunction. This was further supported through hypoxic upregulation of ROCK and ER stress, with amplified effects in TgNotch3_R169C_ mice. Reduced NO/cGMP/PKG (protein kinase G) signaling also promotes PH via ROCK.^33^ In TgNotch_WT_, hypoxia increased ER stress markers (BiP and pIRE1) to levels observed in normoxic TgNotch3_R169C_ mice. Further supporting a role for ER stress in hypoxic TgNotch3_R169C_ mice, 4-PBA rescued vascular reactivity changes. Hypoxia and Notch3 upregulation in TgNotch3_WT_ mice appear to recapitulate aberrant baseline TgNotch3_R169C_ vascular reactivity, and further affect TgNotch3_R169C_ phenotype, via ER stress and ROCK. These findings define functional interplay between Notch3, ROCK, and oxidative and ER stress in a hypoxic PH context.

Molecular processes underlying pulmonary vascular hypereactivity during Notch3 activation focused on procontractile signaling pathways including Ca^2+^-dependent and Ca^2+^-independent mechanisms. Contraction and Ca^2+^ transients in TgNotch3^R169C^ arteries and PASMCs were enhanced in response to 2 ligands, suggesting that altered vascular reactivity is a generalized phenomenon. In line with this, expression of membrane-associated Ca^2+^ channels (Cav1.1, Cav3.1) and subcellular Ca^2+^ stores (RyR, IP3R) were increased. Notch3 was previously implicated in regulating store-operated Ca^2+^ entry through transient receptor potential cation 6 channels in PASMCs, a process enhanced by hypoxia.^34^ Dysregulation of ER Ca^2+^ homeostasis can also promote ER stress, linked to Notch3 signaling. ER stress stimulates the unfolded protein response, which may contribute to deposition of granular osmiophilic material, a hallmark feature of small vessels in CADASIL recapitulated in TgNotch3_R169C_.^7,8^ Our molecular data demonstrate increases in BiP and IRE1 phosphorylation. Notch3 overexpression in cell lines upregulates BiP,^16^ and during ER stress, Notch3ICD has been shown to promote IRE1α activation via BiP interaction.^35^ Functionally, we confirmed that reducing ER stress with chaperone 4-PBA restores TgNotch3_R169C_ pulmonary artery dysfunction and Ca^2+^ transients. Our findings are consistent with Ca^2+^-dependent and ER stress-induced VSMC contraction.^36^ A lack of strain difference in KCl-induced contraction supports our proposal that GOF Notch3 promotes hypercontractility primarily via intracellular Ca^2+^ alterations, rather than voltage-gated effects at the membrane.

Ca^2+^-independent regulation of vascular contraction was explored by probing ROCK pathways. Altered TgNotch3^R169C^ pulmonary artery reactivity was associated with upregulation of Rho-GEFs PDZ and LARG, which enhance ROCK-mediated vascular contraction.^37^ To confirm a role for ROCK in Notch3-associated vascular hyperreactivity, we showed that ROCK inhibition attenuated vascular contraction in hypoxic Notch3 mice. Of relevance, Notch signals through ROCK,^38^ and RhoA-GTPase is implicated as an ER stress/unfolded protein response modulator.^39^ Together these data link Notch3, ROCK and ER stress.

Redox signaling was also altered in TgNotch3_R169C_ animals. ROS production and markers of oxidative stress were increased in TgNotch3_R169C_ lung, whereas H_2_O_2_ levels were decreased. Expression of Nox4, which produces H_2_O_2_ over O_2_^-,40^ was reduced and may contribute to lower H_2_O_2_ generation in TgNotch3_R169C_ lung. Nox4-derived H_2_O_2_ is an endothelial-derived relaxation factor.^41^ Thus reduced H_2_O_2_ may support reduced vasorelaxation in TgNotch3_R169C_ mice. N-acetylcysteine correction of Ca^2+^ transients in TgNotch3_R169C_ PASMCs further supports ROS influence in vascular contraction in TgNotch3_R169C_ lungs.^42^

Both endothelium-dependent and -independent relaxation were compromised in TgNotch3_R169C_ pulmonary arteries, recapitulating previous results in TgNotch3_R169C_ cerebral arteries^43^ and peripheral vessels in patients with CADASIL.^8^ We examined eNOS signaling as the regulator of endothelial-dependent relaxation but failed to demonstrate altered activity. However, increased ROS and NO can react to produce injurious radical peroxynitrite (ONOO^−^). Decreased acetylcholine-mediated relaxation in TgNotch3_R169C_ arteries accompanied indications of elevated ROS/ONOO^−^ and reduced NO. We propose increased R169C ONOO^-^ is detrimental to pulmonary vasorelaxation through both oxidative stress and reduced NO bioavailability. Together these, findings suggest a prooxidative environment promotes TgNotch3_R169C_ endothelial dysfunction.

Reduced relaxation to SNP, a direct NO donor, indicated VSMC dysfunction in TgNotch3_R169C_ mice. To further explore this we investigated redox-sensitive sGC/cGMP/PKG signaling. sGC oxidation decreases NO-mediated cGMP production.^44^ In TgNotch3_R169C_ animals sGCβ1 sulfenylation was increased, consistent with previous findings.^8^ This was associated with oxidative stress, lower cGMP levels, and diminished PKG activity, processes involved in impaired vasorelaxation. Unlike sGC, H_2_O_2_ oxidation activates PKG independently of cGMP^45^ and contributes to PKG-mediated vasorelaxation.^41^ Lower H_2_O_2_ in TgNotch3_R169C_ may reduce PKG activity. Together, our findings indicate multiple mechanisms are involved in Notch3-induced redox alterations that may influence NO/sGC/PKG signaling.

To explore the significance of our findings in human pathophysiology, we studied PASMCs from clinically phenotyped PAH patients. Elevated Notch3, TMIC fragment, and Hes5 expression in PAH versus non-PAH PASMCs demonstrated an increased Notch3 signaling, recapitulating findings in our R169C mice and other PH models.^9,46^ Upregulation of BiP and ROS generation in PAH PASMCs were also similar to observations in hypoxia-treated TgNotch3_R169C_ mice. Normalization of oxidative stress by secretase inhibitor GSI suggests a role for Notch3 in ROS generation in PAH PASMCs, corroborating our findings in experimental models.

In contrast to findings in the mice, H_2_O_2_ and Nox4 were upregulated in PAH cells as previously described.^47,48^ Differences in Nox4/H_2_O_2_ between mouse and human PH models may indicate the role of endothelial cells in our whole lung approach, where H_2_O_2_ is normally abundant and protective, whereas our human studies examined isolated VSMCs, in which Nox4-derived H_2_O_2_ is injurious.^49^ A potential switch from low-level H_2_O_2_ as a signaling molecule to a damaging ROS at higher levels is also suggested. The exact role of Notch3-regulated Nox4 and H_2_O_2_ in endothelial cells versus VSMCs in PH awaits further clarification.

## PERSPECTIVES

We provide evidence that GOF Notch3 mutation and increased Notch3 signaling promote pulmonary vascular dysfunction and remodeling, through redox-sensitive pathways involving ROCK and Ca^2+^ signaling. These processes are driven by ER and oxidative stress, which also negatively influence the NO/sGC/cGMP relaxation pathway. Hypoxia amplifies these aberrations, predisposing to development of PH. We define a novel Notch3-sensitive molecular mechanism involving redox-regulated procontractile and anti-vasodilatory signaling pathways in PH. This notion places Notch3 upstream of other PH-associated molecular mechanisms. These data provide insights on putative novel candidates in PH therapeutics, an area with unmet needs. Our data are particularly interesting in the context of recent studies validating antibody inhibition of Notch3 as a potential PH treatment,^46^ which could reduce not only established Notch3 proliferative effects but also vascular hyperreactivity and endothelial dysfunction that we have highlighted.

## ARTICLE INFORMATION

### Acknowledgments

The authors thank Dr Anne Joutel at the Institut National de la Santé et de la Recherche Médicale (INSERM) for kindly providing the transgenic TgNotch3 model. The authors also thank Jacqueline Thomson, Laura Haddow, John McAbney, and Wendy Beattie for technical support.

### Sources of Funding

This work was funded by grants from the British Heart Foundation (BHF; RG_F_21_110047) and Medical Research Council (MRC; MR/R502327/1 and MR/T015713/1). A.C. Montezano was supported by a University of Glasgow Walton Fellowship. R.M. Touyz was supported through a BHF Chair award (CH/4/29762), the Dr Phil Gold Chair, McGill University, Montreal, and a Canada Research Chair, Canadian Institutes of Health Research.

### Disclosures

None.

## Supplementary Material

## References

[1] Artavanis-Tsakonas S Rand MD Lake RJ. Notch signaling: cell fate control and signal integration in development. Science. 1999;284:770–776. doi: 10.1126/science.284.5415.770 10221902 10.1126/science.284.5415.770

[2] Baeten J Lilly B. Notch signaling in vascular smooth muscle cells. Adv Pharmacol. 2017;78:351–382. doi: 10.1016/bs.apha.2016.07.002 28212801 10.1016/bs.apha.2016.07.002 PMC5964982

[3] Coupland K Lendahl U Karlström H. Role of NOTCH3 mutations in the cerebral small vessel disease cerebral autosomal dominant arteriopathy with subcortical infarcts and leukoencephalopathy. Stroke. 2018;49:2793–2800. doi: 10.1161/STROKEAHA.118.021560 30355220 10.1161/STROKEAHA.118.021560

[4] Morris H Neves K Montezano A MacLean M Touyz R. Notch3 signalling and vascular remodelling in pulmonary arterial hypertension. Clin Sci (Lond). 2019;133:2481–2498. doi: 10.1042/cs20190835 31868216 10.1042/CS20190835 PMC6928565

[5] Joutel A Corpechot C Ducros A Vahedi K Chabriat H Mouton P Alamowitch S Domenga V Cecillion M Marechal E. Notch3 mutations in CADASIL, a hereditary adult-onset condition causing stroke and dementia. Nature. 1996;383:707–710. doi: 10.1038/383707a0 8878478 10.1038/383707a0

[6] Cognat E Baron-Menguy C Domenga-Denier V Cleophax S Fouillade C Monet-Lepretre M Dewerchin M Joutel A. Archetypal Arg169Cys mutation in NOTCH3 does not drive the pathogenesis in cerebral autosomal dominant arteriopathy with subcortical infarcts and leucoencephalopathy via a loss-of-function mechanism. Stroke. 2014;45:842–849. doi: 10.1161/STROKEAHA.113.003339 24425116 10.1161/STROKEAHA.113.003339

[7] Joutel A Monet-Leprêtre M Gosele C Baron-Menguy C Hammes A Schmidt S Lemaire-Carrette B Domenga V Schedl A Lacombe P. Cerebrovascular dysfunction and microcirculation rarefaction precede white matter lesions in a mouse genetic model of cerebral ischemic small vessel disease. J Clin Invest. 2010;120:433–445. doi: 10.1172/JCI39733 20071773 10.1172/JCI39733 PMC2810078

[8] Neves KB Harvey AP Moreton F Montezano AC Rios FJ Alves-Lopes R Nguyen Dinh Cat A Rocchicciolli P Delles C Joutel A. ER stress and Rho kinase activation underlie the vasculopathy of CADASIL. JCI Insight. 2019;4:e131344. doi: 10.1172/jci.insight.131344 31647781 10.1172/jci.insight.131344 PMC6962020

[9] Li X Zhang X Leathers R Makino A Huang C Parsa P Macias J Yuan JX Jamieson SW Thistlethwaite PA. Notch3 signaling promotes the development of pulmonary arterial hypertension. Nat Med. 2009;15:1289–1297. doi: 10.1038/nm.2021 19855400 10.1038/nm.2021 PMC2780347

[10] Liu X Mei M Chen X Lu Y Dong X Hu L Hu X Cheng G Cao Y Yang L. Identification of genetic factors underlying persistent pulmonary hypertension of newborns in a cohort of Chinese neonates. Respir Res. 2019;20:174. doi: 10.1186/s12931-019-1148-1 31382961 10.1186/s12931-019-1148-1 PMC6683566

[11] Padhye AA Sahay S. An adult case of NOTCH3 mutation in pulmonary artery hypertension. Pulm Circ. 2022;12:e12050. doi: 10.1002/pul2.12050 35506080 10.1002/pul2.12050 PMC9052997

[12] Gustafsson MV Zheng X Pereira T Gradin K Jin S Lundkvist J Ruas JL Poellinger L Lendahl U Bondesson M. Hypoxia requires notch signaling to maintain the undifferentiated cell state. Dev Cell. 2005;9:617–628. doi: 10.1016/j.devcel.2005.09.010 16256737 10.1016/j.devcel.2005.09.010

[13] Takahashi K Adachi K Yoshizaki K Kunimoto S Kalaria RN Watanabe A. Mutations in NOTCH3 cause the formation and retention of aggregates in the endoplasmic reticulum, leading to impaired cell proliferation. Hum Mol Genet. 2010;19:79–89. doi: 10.1093/hmg/ddp468 19825845 10.1093/hmg/ddp468

[14] Neves KB Morris HE Alves-Lopes R Muir KW Moreton F Delles C Montezano AC Touyz RM. Peripheral arteriopathy caused by Notch3 gain-of-function mutation involves ER and oxidative stress and blunting of NO/sGC/cGMP pathway. Clin Sci (Colch). 2021;135:753–773. doi: 10.1042/cs20201412 33681964 10.1042/CS20201412

[15] Koyama M Furuhashi M Ishimura S Mita T Fuseya T Okazaki Y Yoshida H Tsuchihashi K Miura T. Reduction of endoplasmic reticulum stress by 4-phenylbutyric acid prevents the development of hypoxia-induced pulmonary arterial hypertension. Am J Physiol Heart Circ Physiol. 2014;306:H1314–H1323. doi: 10.1152/ajpheart.00869.2013 24610918 10.1152/ajpheart.00869.2013

[16] Chida A Shintani M Matsushita Y Sato H Eitoku T Nakayama T Furutani Y Hayama E Kawamura Y Inai K. Mutations of NOTCH3 in childhood pulmonary arterial hypertension. Mol Genet Genomic Med. 2014;2:229–239. doi: 10.1002/mgg3.58 24936512 10.1002/mgg3.58 PMC4049363

[17] Shimokawa H Sunamura S Satoh K. RhoA/Rho-kinase in the cardiovascular system. Circ Res. 2016;118:352–366. doi: 10.1161/CIRCRESAHA.115.306532 26838319 10.1161/CIRCRESAHA.115.306532

[18] de Chantemèle EB Retailleau K Pinaud F Vessières E Bocquet A Guihot A Lemaire B Domenga V Baufreton C Loufrani L. Notch3 is a major regulator of vascular tone in cerebral and tail resistance arteries. Arterioscler Thromb Vasc Biol. 2008;28:2216–2224. doi: 10.1161/atvbaha.108.171751 18818417 10.1161/ATVBAHA.108.171751 PMC2658748

[19] Camargo LL Harvey AP Rios FJ Tsiropoulou S de Oliveira Silva RDN Cao Z Graham D McMaster C Burchmore RJ Hartley RC. Vascular Nox compartmentalization, protein hyperoxidation and ER stress response in hypertension. Hypertension. 2018;72:235–246. doi: 10.1161/hypertensionaha.118.10824 29844144 10.1161/HYPERTENSIONAHA.118.10824 PMC6004120

[20] Delbrel E Soumare A Naguez A Label R Bernard O Bruhat A Fafournoux P Tremblais G Marchant D Gille T. HIF-1α triggers ER stress and CHOP-mediated apoptosis in alveolar epithelial cells, a key event in pulmonary fibrosis. Sci Rep. 2018;8:17939. doi: 10.1038/s41598-018-36063-2 30560874 10.1038/s41598-018-36063-2 PMC6299072

[21] Siques P Brito J Pena E. Reactive oxygen species and pulmonary vasculature during hypobaric hypoxia. Front Physiol. 2018;9:865. doi: 10.3389/fphys.2018.00865 30050455 10.3389/fphys.2018.00865 PMC6052911

[22] Baron-Menguy C Domenga-Denier V Ghezali L Faraci FM Joutel A. Increased Notch3 activity mediates pathological changes in structure of cerebral arteries. Hypertension. 2017;69:60–70. doi: 10.1161/HYPERTENSIONAHA.116.08015 27821617 10.1161/HYPERTENSIONAHA.116.08015 PMC5145742

[23] Ghezali L Capone C Baron-Menguy C Ratelade J Christensen S Ostergaard Pedersen L Domenga-Denier V Pedersen JT Joutel A. Notch3(ECD) immunotherapy improves cerebrovascular responses in CADASIL mice. Ann Neurol. 2018;84:246–259. doi: 10.1002/ana.25284 30014602 10.1002/ana.25284

[24] Keegan A Morecroft I Smillie D Hicks MN MacLean MR. Contribution of the 5-HT(1B) receptor to hypoxia-induced pulmonary hypertension: converging evidence using 5-HT(1B)-receptor knockout mice and the 5-HT(1B/1D)-receptor antagonist GR127935. Circ Res. 2001;89:1231–1239. doi: 10.1161/hh2401.100426 11739290 10.1161/hh2401.100426

[25] White K Johansen AK Nilsen M Ciuclan L Wallace E Paton L Campbell A Morecroft I Loughlin L McClure JD. Activity of the estrogen-metabolizing enzyme cytochrome P450 1B1 influences the development of pulmonary arterial hypertension. Circulation. 2012;126:1087–1098. doi: 10.1161/CIRCULATIONAHA.111.062927 22859684 10.1161/CIRCULATIONAHA.111.062927

[26] Montezano AC Lopes RA Neves KB Rios F Touyz RM. Isolation and culture of vascular smooth muscle cells from small and large vessels. Methods Mol Biol. 2017;1527:349–354. doi: 10.1007/978-1-4939-6625-7_27 28116729 10.1007/978-1-4939-6625-7_27

[27] Hurst LA Dunmore BJ Long L Crosby A Al-Lamki R Deighton J Southwood M Yang X Nikolic MZ Herrera B. TNFα drives pulmonary arterial hypertension by suppressing the BMP type-II receptor and altering NOTCH signalling. Nat Commun Nature. 2017;8:14079. doi: 10.1038/ncomms14079 10.1038/ncomms14079 PMC5241886 28084316

[28] Alves-Lopes R Neves KB Anagnostopoulou A Rios FJ Lacchini S Montezano AC Touyz RM. Crosstalk between vascular redox and calcium signaling in hypertension involves TRPM2 (Transient Receptor Potential Melastatin 2) cation channel. Hypertension. 2020;75:139–149. doi: 10.1161/HYPERTENSIONAHA.119.13861 31735084 10.1161/HYPERTENSIONAHA.119.13861

[29] Ikeda K Hale P Pauciulo M Dasgupta N Pastura P Le Cras T Pandey M Nichols W. Hypoxia-induced pulmonary hypertension in different mouse strains: relation to transcriptome. Am J Respir Cell Mol Biol. 2019;60:106–116. doi: 10.1165/rcmb.2017-0435OC 30134121 10.1165/rcmb.2017-0435OC PMC6348717

[30] Noseda M Fu Y Niessen K Wong F Chang L McLean G Karsan A. Smooth Muscle alpha-actin is a direct target of Notch/CSL. Circ Res. 2006;98:1468–1470. doi: 10.1161/01.RES.0000229683.81357.26 16741155 10.1161/01.RES.0000229683.81357.26

[31] Wang LL Zhu XL Han SH Xu L. Hypoxia upregulates NOTCH3 signaling pathway to promote endothelial-mesenchymal transition in pulmonary artery endothelial cells. Evid Based Complement Alternat Med. 2021;2021:1525619. doi: 10.1155/2021/1525619 34868328 10.1155/2021/1525619 PMC8639273

[32] Jaitovich A Jourd’heuil D. A brief overview of nitric oxide and reactive oxygen species signaling in hypoxia-induced pulmonary hypertension. Adv Exp Med Biol. 2017;967:71–81. doi: 10.1007/978-3-319-63245-2_6 29047082 10.1007/978-3-319-63245-2_6 PMC5863727

[33] Zhao Y Cai L Mirza M Huang X Geenen D Hofmann F Yuan J Zhao Y. Protein kinase G-I deficiency induces pulmonary hypertension through Rho A/Rho kinase activation. Am J Pathol. 2012;180:2268–2275. doi: 10.1016/j.ajpath.2012.02.016 22632818 10.1016/j.ajpath.2012.02.016 PMC3717780

[34] Smith KA Voiriot G Tang H Fraidenburg DR Song S Yamamura H Yamamura A Guo Q Wan J Pohl NM. Notch activation of Ca(2+) signaling in the development of hypoxic pulmonary vasoconstriction and pulmonary hypertension. Am J Respir Cell Mol Biol. 2015;53:355–367. doi: 10.1165/rcmb.2014-0235OC 25569851 10.1165/rcmb.2014-0235OC PMC4566064

[35] Giuli MV Diluvio G Giuliani E Franciosa G Di Magno L Pignataro MG Tottone L Nicoletti C Besharat ZM Peruzzi G. Notch3 contributes to T-cell leukemia growth via regulation of the unfolded protein response. Oncogenesis. 2020;9:93. doi: 10.1038/s41389-020-00279-7 33071287 10.1038/s41389-020-00279-7 PMC7569087

[36] Liang B Wang S Wang Q Zhang W Viollet B Zhu Y Zou M. Aberrant endoplasmic reticulum stress in vascular smooth muscle increases vascular contractility and blood pressure in mice deficient of AMP-activated protein kinase-α2 in vivo. Arterioscler Thromb Vasc Biol. 2013;33:595–604. doi: 10.1161/ATVBAHA.112.300606 23288166 10.1161/ATVBAHA.112.300606 PMC3754846

[37] Wynne B Chiao C Webb R. Vascular smooth muscle cell signaling mechanisms for contraction to angiotensin II and endothelin-1. J Am Soc Hypertens. 2009;3:84–95. doi: 10.1016/j.jash.2008.09.002 20161229 10.1016/j.jash.2008.09.002 PMC2704475

[38] Venkatesh D Fredette N Rostama B Tang Y Vary C Liaw L Urs S. RhoA-mediated signaling in Notch-induced senescence-like growth arrest and endothelial barrier dysfunction. Arterioscler Thromb Vasc Biol. 2011;31:876–882. doi: 10.1161/ATVBAHA.110.221945 21273559 10.1161/ATVBAHA.110.221945 PMC3252819

[39] Kawanami D Matoba K Okada R Tsukamoto M Kinoshita J Ishizawa S Kanazawa Y Yokota T Utsunomiya K. Fasudil inhibits ER stress-induced VCAM-1 expression by modulating unfolded protein response in endothelial cells. Biochem Biophys Res Commun. 2013;435:171–175. doi: 10.1016/j.bbrc.2013.04.091 23665024 10.1016/j.bbrc.2013.04.091

[40] Nisimoto Y Diebold B Cosentino-Gomes D Lambeth J. Nox4: a hydrogen peroxide-generating oxygen sensor. Biochemistry. 2014;53:5111–5120. doi: 10.1021/bi500331y 25062272 10.1021/bi500331y PMC4131900

[41] Shimokawa H. Hydrogen peroxide as an endothelium-derived hyperpolarizing factor. Pflugers Arch. 2010;459:915–922. doi: 10.1007/s00424-010-0790-8 20140449 10.1007/s00424-010-0790-8

[42] Jernigan N Walker B Resta T. Reactive oxygen species mediate RhoA/Rho kinase-induced Ca2+ sensitization in pulmonary vascular smooth muscle following chronic hypoxia. Am J Physiol Lung Cell Mol Physiol. 2008;295:L515–L529. doi: 10.1152/ajplung.00355.2007 18621909 10.1152/ajplung.00355.2007 PMC2536792

[43] Capone C Cognat E Ghezali L Baron-Menguy C Aubin D Mesnard L Stöhr H Domenga-Denier V Nelson M Joutel A. Reducing Timp3 or vitronectin ameliorates disease manifestations in CADASIL mice. Ann Neurol. 2016;79:387–403. doi: 10.1002/ana.24573 26648042 10.1002/ana.24573 PMC5359978

[44] Shah R Sanker S Wood K Durgin B Straub A. Redox regulation of soluble guanylyl cyclase. Nitric Oxide. 2018;76:97–104. doi: 10.1016/j.niox.2018.03.013 29578056 10.1016/j.niox.2018.03.013 PMC5916318

[45] Sheehe J Bonev A Schmoker A Ballif B Nelson M Moon T Dostmann W. Oxidation of cysteine 117 stimulates constitutive activation of the type Iα cGMP-dependent protein kinase. J Biol Chem. 2018;293:16791–16802. doi: 10.1074/jbc.RA118.004363 30206122 10.1074/jbc.RA118.004363 PMC6204908

[46] Zhang Y Hernandez M Gower J Winicki N Morataya X Alvarez S Yuan JX Shyy J Thistlethwaite PA. JAGGED-NOTCH3 signaling in vascular remodeling in pulmonary arterial hypertension. Sci Transl Med. 2022;14:eabl5471. doi: 10.1126/scitranslmed.abl5471 35507674 10.1126/scitranslmed.abl5471

[47] Hood KY Montezano AC Harvey AP Nilsen M MacLean MR Touyz RM. Nicotinamide adenine dinucleotide phosphate oxidase-mediated redox signaling and vascular remodeling by 16alpha-hydroxyestrone in human pulmonary artery cells: implications in pulmonary arterial hypertension. Hypertension. 2016;68:796–808. doi: 10.1161/HYPERTENSIONAHA.116.07668 27402919 10.1161/HYPERTENSIONAHA.116.07668 PMC4978604

[48] Hood KY Mair KM Harvey AP Montezano AC Touyz RM MacLean MR. Serotonin signaling through the 5-HT1B receptor and NADPH oxidase 1 in pulmonary arterial hypertension. Arterioscler Thromb Vasc Biol. 2017;37:1361–1370. doi: 10.1161/ATVBAHA.116.308929 28473438 10.1161/ATVBAHA.116.308929 PMC5478178

[49] Touyz RM Montezano AC. Vascular Nox4: a multifarious NADPH oxidase. Circ Res. 2012;110:1159–1161. doi: 10.1161/CIRCRESAHA.112.269068 22539753 10.1161/CIRCRESAHA.112.269068

