# Supplementary material for: Notch3/Hes5 Induces Vascular Dysfunction in Hypoxia-Induced Pulmonary Hypertension Through ER Stress and Redox-Sensitive Pathways
Source: Hypertension. 2023 May 31;80(8):1683–96. doi: 10.1161/HYPERTENSIONAHA.122.20449 (PMC10355806; doi:10.1161/HYPERTENSIONAHA.122.20449)
Supplement: Supplementary file 1 [file hyp-80-1683-s001.pdf]

ONLINE SUPPLEMENT

Notch3/Hes5 induces vascular dysfunction in hypoxia-induced pulmonary hypertension through ER stress and redox-sensitive pathways

Hannah E Morris PhD.[1], Karla B Neves PhD.[1], Margaret Nilsen[2], Augusto C Montezano PhD.[1], Margaret R MacLean PhD.[2], *Rhian M Touyz MD., PhD.[1,3].

[1]Institute of Cardiovascular and Medical Sciences, University of Glasgow, UK; [2]Strathclyde Institute of Pharmacy and Biomedical Sciences, University of Strathclyde, UK; [3]Research Institute of McGill University Health Centre, McGill University, Canada

Short title: Notch3 and pulmonary hypertension

Corresponding author:

Rhian M Touyz MD, PhD

Research Institute of McGill University Health Centre,

McGill University,

1001 Boulevard Decarie

Montreal,

Canada

Tel: +1 514 934 1934 x 71608

Rhian Touyz (rhian.touyz@mcgill.ca)

Detailed methods

Notch3 mutant mouse model

All experimental procedures were conducted in accordance with the UK Animals (Scientific Procedures) Act 1986, ARRIVE guidelines, and local institutional guidelines. All experiments were conducted by experienced researchers and analysis was carried out in a blinded fashion. The transgenic (Tg) mouse lines, $TgNotch3_{WT}$ and $TgNotch3_{R169C}$ have been described and characterised previously (Joutel et al. 2010). Mice overexpress either a WT or R169C mutant sequence rat Notch3 gene; overexpression is ubiquitous and not SMC-specific, but characterisation of the model has shown an endogenous expression pattern of the transgene in the medial layer and pericytes (Joutel et al. 2010). Our earlier work demonstrated that the R169C mutation has gain-of-function effects through increased Notch3 signalling in peripheral VSMC and arteries, affecting vascular reactivity and vascular signalling pathways; however, systolic blood pressures of $TgNotch3_{R169C}$ mice were not raised compared to wild-type ($TgNotch3_{WT}$) controls (Neves et al., 2019). Male mice were housed at a controlled humidity and temperature (22°C - 24°C) in light/dark cycles of 12 hours and with access to food and water *ad libitum*. Previous work has demonstrated this model and others exhibit typical features of human CADASIL by 6 months of age (Joutel et al., 2010; Baron-Menguy et al., 2017), therefore mice in these experiments were used at this age. Only male mice of this model were used as females are thought to exhibit mosaic transgene expression (Ghezali et al. 2018) and do no exhibit alterations to pulmonary vascular reactivity (unpublished data). Lung tissue was collected for molecular experiments. Intralobar pulmonary arteries were used to assess vascular function using myography and isolation of smooth muscle cells for further experiments.

Pulmonary vascular functional studies by wire myography

Intralobar pulmonary arteries (third order; ~350µm internal diameter) were isolated from $TgNotch3_{WT}$ and $TgNotch3_{R169C}$ mice for wire myography as previously described (White et al. 2012). Briefly, intra-lobar arteries were dissected out of surrounding tissue from the left lung and sectioned into arterial rings (~1.8 - 2mm). These were mounted on isometric wire myograph baths (Danish Myo Technology, Denmark) within 5ml of physiological saline solution [(in mmol/L: 130 NaCl, 14.9 $NaHCO_3$, 4.7 KCl, 1.18KH_2PO_4, 1.17 $MgSO_4.7H_2O$, 5.5 glucose, 1.56 $CaCl_2.2H_2O$, and 0.026 EDTA]. Solutions were maintained at a constant temperature of 37±0.5ºC and were continuously gassed with a mixture of 16% O_2, 5% CO_2 (balance N_2). The specific gas mixture was used mimic the partial pressures of gases in the pulmonary arteries, which carry less oxygenated blood. Arteries from normoxic animals were normalised to 2.2kPa, equivalent to a normal pulmonary artery transmural pressure of ~16.5mmHg. Arteries from hypoxic animals were normalised to 4kPa (30 to 33 mmHg) (Keegan et al., 2001). Following 30 minutes equilibration, contractile responses of arterial segments were assessed with the addition of KCl (62.5mmol/L). Any vessels that did not respond with at least 0.8mN of tension following KCl were excluded. Functioning endothelium was verified by relaxation induced by a single dose of ACh ($3x10^{-6}$ mol/L) in arteries pre-contracted with U46619 ($3x10^{-8}$ mol/L). Cumulative concentration response curves were constructed to 5-hydroxytryptamine (5-HT, 10^{-9} – 10^{-5} mol/L) and endothelin-1 (ET-1, 10^{-12} – 10^{-8} mol/L) to evaluate vasoconstriction. Endothelium-dependent relaxation was assessed as a dose-response to ACh (10^{-9} - 10^{-5} mol/L) and endothelium-independent vasorelaxation was assessed by a dose-response to SNP (10^{-9} – 10^{-5} mol/L). Relaxation and contraction responses were also assessed in the presence of ROCK inhibitor fasudil (1 µmol/L, 45 minutes) or ER stress inhibitor 4-84 phenylbutyrate (4-PBA; 1 mmol/L, 45 minutes). Recording and analysis was performed in LabChart 8 Reader (ADInstruments; Dunedin, New Zealand).

PASMC isolation from mouse lung

PASMCs from non-Tg FVB littermates, $TgNotch3_{WT}$ and $TgNotch3_{R169C}$ animals (mPASMCs) were isolated from third order intralobar pulmonary arteries. Methods for mouse VSMC isolation and culture are previously described (Montezano et al., 2017). Briefly, cleaned arteries were placed in Ham's F-12 culture medium containing 1% gentamicin, collagenase (type 1), elastase, soybean trypsin inhibitor and BSA, and were incubated for 30 to 60 minutes at 37°C under constant agitation. Digested arteries were then repeatedly aspirated through a 20G needle for further breakdown. The suspension was centrifuged (2000 rpm, 3 minutes) and the cell pellet was resuspended in high glucose (4.5g/L) DMEM media supplemented with 10% fetal bovine serum (FBS) and Penicillin/Streptomycin (50 µg/ml) and Amphoterecin B (0.5 µg/ml). Cells were seeded into 25mm flasks and maintained in the same media. Before experimentation, cells were quiesced overnight using reduced growth supplement medium (0.5% FBS). Cells were used for experiments between passages 3 and 8. In some protocols, the role of ROCK, ER stress, and Notch signalling was assessed with pharmacological inhibitors: ROCK inhibitor fasudil (1 µmol/L, Tocris, UK), ER stress inhibitor and chemical chaperone 4-phenylbutyric acid (4-PBA) (1 mmol/L, Sigma-Aldrich, UK), and Notch inhibitor gamma secretase inhibitor (GSI) XXIII (5 µmol/L, Calbiochem, San Diego). Cells were pre-treated with inhibitors for 24 hours.

Isolation and Culture of human PASMCs

Human pulmonary artery smooth muscle cells (hPASMCs) were isolated from pulmonary arteries (0.3 – 1 mm diameter) from distal portions of macroscopically normal lung tissue from patients undergoing pneumonectomy, or from lungs of PAH patients undergoing lung transplantation or at autopsy. All human cells were isolated in Professor Nicholas Morrell's laboratory, Cambridge, UK as described previously (Hurst et al., 2017). All studies on human tissue were approved by an institutional review committee (REC ref. 18/EE/0269), and studies conformed to local and national guidelines. Patient characteristics can be found in the supplemental data.

Measurement of intracellular Ca^{2+} transients in mPASMCs

Intracellular free Ca^{2+} concentration ($[Ca^{2+}]_i$) was measured in PASMCs from FVB, $TgNotch3_{WT}$ and $TgNotch3_{R169C}$ mice using the fluorescent Ca^{2+} indicator, Cal-520 acetoxymethyl ester (Cal-520/AM; Abcam; 10 µmol/L) as previously described (Alves-Lopes et al., 2020). Isolated mPASMCs were grown in 6-well plates before removal of maintenance media and addition of Cal-520 AM in 0.5% FBS DMEM. Incubation was for 75 minutes at 37°C, followed with 30 minutes at room temperature, in dark conditions throughout. Following incubation, the dye solution was replaced with HEPES physiological saline solution ($1.3x10^{-1}$ mol/L NaCl, $5x10^{-3}$ mol/L KCl, 10^{-3} mol/L CaCl, 10^{-3} mol/L MgCl, $2x10^{-2}$ mol/L HEPES, and 10^{-2} mol/L D-glucose, pH 7.4) for 30 minutes prior to imaging. Fluorescence intensity as a measure of $[Ca^{2+}]$i, was monitored for 30 seconds in basal condition before stimulation for 240 seconds with 5-HT (1 µmol/L) or ET-1 (100 nmol/L) stimulation. In some experiments, PASMCs were pre-treated with 4-PBA or GSI for before stimulation. Fluorescence-based measurements of Ca^{2+} signals were performed using an inverted epifluorescence microscope (Axio Observer Z1 Live-Cell imaging system; Zeiss, Cambridge, UK) with excitation/emission wavelengths 490/535nm, respectively. Images were acquired and analysed using Zen Blue software (Zeiss, Cambridge, UK). Area under the curve (AUC) was determined from the curve for each group and represents the percentage change in Ca^{2+} intensity over the full 240 seconds of data collection. AUC was used only for statistical purposes.

Chronic hypoxic studies

In a separate cohort of animals, the development of PH was achieved with 14 days chronic hypobaric hypoxia as described previously (Keegan et al., 2001). Briefly, 24-week male animals were housed for 14 days in a hypobaric chamber simulating low atmospheric oxygen (550mbar/10% O_2) to induce hypoxic pulmonary vasoconstriction and subsequent pulmonary vascular remodelling. Animals were in hypoxic condition throughout, with the exception of ~2hr per week for animal husbandry. Normoxic age-matched littermates maintained in room air were studied as controls.

Hemodynamic Measurements

Animals were anaesthetically induced in 3% (v/v) isoflurane, then maintained at approximately 1.5% (v/v) isoflurane supplemented with a constant flow of medical oxygen (1L/min). Haemodynamic parameters were recorded using a Millar PVR-1030 catheter. The catheter was placed into the right jugular vein and advanced into the right ventricle, as described previously (Keegan et al., 2001) to assess right ventricular systolic pressure (RVSP). Mean systemic arterial pressure was assessed by placement of the catheter in the right carotid artery and left ventricle. Recordings were made and analysed using the corresponding software (LabChart Pro version 8, ADInstruments; Dunedin, New Zealand) as described previously.

Right Ventricular Hypertrophy

To assess right ventricular hypertrophy, the right ventricular free wall was separated and weighed. As previously described, this was then expressed as a ratio to the left ventricular wall plus septum weight (RV/(LV+S)) (Keegan et al., 2001).

Lung Histopathology and Immunohistochemistry

Lung sagittal sections (5 µm) from $TgNotch3_{WT}$ and $TgNotch3_{R169C}$ mice were stained with Miller's elastin/PicroSirius Red. Pulmonary arteries <80 µm external diameter were then microscopically assessed in a blinded fashion to assess vascular remodelling. The number of remodelled vessels (indicated by a distinctive double elastic lamina) was expressed over the total number of vessels present in a lung section. Vessels were classed as non-remodelled, partially remodelled (25-50% double elastic lamina) or fully remodelled (>50%). Remodelling of the medial layer in distal pulmonary arteries was also assessed by immune staining for alpha smooth muscle actin (α-SMA), as described previously (White et al., 2012). Sagittal sections (5µm) of lung tissue from $TgNotch3_{WT}$ and $TgNotch3_{R169C}$ mice were mounted on poly-L-lysine slides and firstly underwent antigen retrieval and blocking, before overnight incubation at 4°C with α-SMA (1:200; Abcam, UK) in 1% BSA PBS. After incubation with HRP-conjugated secondary, α-SMA was visualised by DAB (3,3′-Diaminobenzidine) reaction. Sections were counterstained with haematoxylin. Semi-quantitative medial thickness measurements of α-SMA staining were made using Zen2 Core v2.5 software (Zeiss; Cambridge, UK). This was performed in biological triplets; 6 vessels were analysed from each section using three sequential sections from each animal. An average was taken of two vessel wall thickness measurements per vessel and expressed as a percentage of the total vessel diameter (TVD).

Quantitative real-time Polymerase Chain Reaction

Gene expression was assessed by quantitative real-time Polymerase Chain Reaction (qPCR) (Qiagen, UK). Briefly, total RNA was extracted from whole lung of $TgNotch3_{WT}$ and $TgNotch3_{R169C}$ mice using QIAzol (Qiagen, Manchester, UK), treated with RNase-free DNAse I, and 2 µg of RNA was reverse transcribed in a reaction containing 100 µg/mL oligo-dT, 10 mmol/L of 2′-deoxynucleoside 5′-triphosphate, 5×first-Strand buffer, and 2 µL of 200-U reverse transcriptase. For real-time PCR amplification, 3 µL of each reverse transcription product were diluted in a reaction buffer containing 5 µL of SYBR Green PCR master mix and 300 nmol/L of primers in a final volume of 10 µL per sample. The reaction conditions were as follows; 2 steps at 50°C for 2 minutes and 95°C for 2 minutes, followed by 40 cycles of 3 steps, 15-second denaturation at 95°C, 60-second annealing at 60°C, and 15 seconds at 72°C. Mouse primers are detailed in supplementary data (Table S1). Data are expressed as target gene/GAPDH reference gene. Relative gene expression was calculated using the $2^{-\Delta\Delta Ct}$ fold change method.

Immunoblotting

Protein was extracted from whole lung from $TgNotch3_{WT}$ and $TgNotch3_{R169C}$ mice. Total protein was determined by BCA assay and equal concentrations of lung lysate (30µg) were loaded on a 4-20% gradient polyacrylamide gel and separated by electrophoresis, then transferred to nitrocellulose membrane. Non-specific binding was blocked with 3% bovine serum albumin in Tris-buffered saline (TBS) solution. Membranes were then incubated with specific primary antibodies overnight at 4°C. Membranes were washed 3 times with TBS-Tween20 and incubated with infrared dye-labelled secondary antibodies for 1 hour at room temperature. Membranes were visualized using an Odyssey CLx infrared imaging system (LiCor Biosciences UK Ltd, UK) and densitometric results were normalized to α-tubulin protein and are expressed in arbitrary units. Antibodies used were as follows: anti-α-tubulin (1:10000; Abcam, UK); anti-Notch3 (1:2000, Cell Signaling Technology); anti-Hes5 (1:500, Santa Cruz Biotechnology); anti-ROCK2 (1:500, BD Biosciences); anti-ROCK1 (1:500; Chemicon International); anti-phospho-$eNOS_{Thr495}$ (1:1000, Cell Signaling Technology), anti-phospho-$eNOS_{Ser1177}$ (1:1000; Cell Signaling Technology); anti-total eNOS (1:1000, Santa Cruz Biotechnology) anti-CHOP (1:500, Santa Cruz Biotechnology); anti-BiP/GRP78 (1:1000, Santa Cruz Biotechnology), anti-IRE1α (1:200, Santa Cruz Biotechnology), anti-phospho-$IRE1\alpha_{Ser724}$ (Cell Signaling Technology), and anti-sGCB1 (1:500, Cayman Chemical).

Lucigenin-enhanced chemiluminescence

Lucigenin-derived chemiluminescence assay was used to determine nicotinamide adenine dinucleotide phosphate (NADPH)-dependent ROS production in whole lung homogenates from $TgNotch3_{WT}$ and $TgNotch3_{R169C}$ mice. Briefly, tissues were homogenized in lysis buffer (20 mmol/L of KH_2PO_4, 1 mmol/L of EGTA, 1 µg/mL of aprotinin, 1 µg/mL of leupeptin, 1 µg/mL of pepstatin, and 1 mmol/L of PMSF). 50 µl of sample was added to 175 µl of assay buffer (50 mmol/L of KH_2PO_4, 1 mmol/L of EGTA, and 150 mmol/L of sucrose) and lucigenin (5 µmol/L). Luminescence was measured by luminometer (AutoLumat LB 953, Berthold) at baseline and after stimulation with NADPH (100 µmol/l). A buffer blank was subtracted from each reading. ROS production was normalised to protein concentration, determined by BCA assay. This method is accepted as preferential for superoxide (O_2^-) with minimal redox cycling of the probe at this concentration.

Amplex Red assay

Hydrogen peroxide (H_2O_2) was assessed in whole lung homogenates from $TgNotch3_{WT}$ and $TgNotch3_{R169C}$ mice using an Amplex Red® Hydrogen Peroxide/Peroxidase fluorescence assay (Life Technologies; Carlsbad, USA) according to the manufacturer's instructions. Fluorescence readings were made in a 96-well plate at Ex/Em = 530/590 nm. H_2O_2 production was normalized to protein concentration, determined by BCA assay.

3-Nitrotyrosine Enzyme-Linked Immunosorbent Assay

The reaction of superoxide and nitric oxide produces $ONOO^-$ causing nitration of tyrosine residues to produce 3NT, which can be used as a biomarker for $ONOO^-$. A competitive ELISA for 3NT (Nitrotyrosine ELISA Kit; Abcam, UK) was used to assess nitrosative stress-mediated 3NT modifications to proteins in whole lung homogenates from $TgNotch3_{WT}$ and $TgNotch3_{R169C}$ according to manufacturer instructions. Samples were solubilised in extraction buffer and total protein concentration was determined by BCA assay. Assays were performed with 3NT-BSA standards (31.25 – 2000 ng/ml). Standards and samples were pipetted into the microplate precoated with 3NT antibody followed by HRP Detector Antibody. After 2 hours incubation at room temperature, the plate was washed, and the reaction terminated. Absorbance measurement was read at 450 nm. 3NT concentration (ng/ml) was extrapolated from the standard curve and corrected for total protein.

Total NOx Assay Kit

Total NO was assessed using a colorimetric kit that detects nitrate and nitrate as the stable end products of NO metabolism (Total NO Detection Kit; Enzo Life Sciences, UK). Whole lung was homogenised in PBS and protein concentration was determined by BCA assay. For each sample 50µg of total protein was loaded into a microtiter plate alongside nitrate standards (3.125 - 100 µmol/L). The assay was then performed according to the manufacturer's instructions. Nitrate in the samples and standards was firstly converted to nitrite with a nitrate reductase step, then nitrite was measured by the Griess reaction. Absorbance was read at 560nm. Total NOx was then extrapolated from the nitrate standard curve.

Affinity capture of sulfenylated proteins

Sulfenylated proteins were assessed by affinity capture using the DCP-Bio1 probe and streptavidin bead protocol, as per manufacturers instructions (Merck; Dorset, UK). Western blot was then performed using the eluted sulfenylated proteins with primary antibodies for sGCβ1 and PKG-1. Biotinylated-Trx Loading Control Protein was used as a control for protein level (Kerafast; Boston, USA). 3 animals were pooled per sample to concentrate protein.

Statistical analysis

All data are expressed as mean±SEM. Comparisons were performed using unpaired two-tailed t-test or one-way ANOVA with Bonferroni *post-hoc* analyses (as appropriate and indicated in figure legends). $p<0.05$ was considered statistically significant. Data analysis and graphing was performed using GraphPad Prism 8.0 (GraphPad Software Inc.; San Diego, USA).

Supplemental References

Alves-Lopes, R., Neves, K. B., Anagnostopoulou, A., Rios, F. J., Lacchini, S., Montezano, A. C., & Touyz, R. M. (2020). Crosstalk Between Vascular Redox and Calcium Signaling in Hypertension Involves TRPM2 (Transient Receptor Potential Melastatin 2) Cation Channel. Hypertension (Dallas, Tex. : 1979), 75(1), 139–149.

Baron-Menguy, C., Domenga-Denier, V., Ghezali, L., Faraci, F. M. & Joutel, A. 2017. Increased Notch3 Activity Mediates Pathological Changes in Structure of Cerebral Arteries. Hypertension, 69, 60-70.

Ghezali, L., Capone, C., Baron-Menguy, C., Ratelade, J., Christensen, S., Ostergaard Pedersen, L., Domenga-Denier, V., Pedersen, J. T. & Joutel, A. 2018. Notch3(ECD) immunotherapy improves cerebrovascular responses in CADASIL mice. Ann Neurol, 84, 246-259.

Hurst, L. A., Dunmore, B. J., Long, L., Crosby, A., Al-Lamki, R., Deighton, J., Southwood, M., Yang, X., Nikolic, M. Z., Herrera, B., Inman, G. J., Bradley, J. R., Rana, A. A., Upton, P. D. & Morrell, N. W. 2017. TNFalpha drives pulmonary arterial hypertension by suppressing the BMP type-II receptor and altering NOTCH signalling. Nat Commun, 8, 14079.

Joutel, A., Monet-Leprêtre, M., Gosele, C., Baron-Menguy, C., Hammes, A., Schmidt, S., Lemaire-Carrette, B., Domenga, V., Schedl, A., Lacombe, P. & Hubner, N. 2010. Cerebrovascular dysfunction and microcirculation rarefaction precede white matter lesions in a mouse genetic model of cerebral ischemic small vessel disease. J Clin Invest, 120, 433-45.

Keegan, A., Morecroft, I., Smillie, D., Hicks, M. N. & Maclean, M. R. 2001. Contribution of the 5-HT(1B) receptor to hypoxia-induced pulmonary hypertension: converging evidence using 5-HT(1B)-receptor knockout mice and the 5-HT(1B/1D)-receptor antagonist GR127935. Circ Res, 89, 1231-9.

Montezano, A. C., Lopes, R. A., Neves, K. B., Rios, F. & Touyz, R. M. 2017. Isolation and Culture of Vascular Smooth Muscle Cells from Small and Large Vessels. Methods Mol Biol, 1527, 349-354.

Neves, K. B., Harvey, A. P., Moreton, F., Montezano, A. C., Rios, F. J., Alves-Lopes, R., Nguyen Dinh Cat, A., Rocchicciolli, P., Delles, C., Joutel, A., Muir, K. & Touyz, R. M. 2019. ER stress and Rho kinase activation underlie the vasculopathy of CADASIL. JCI Insight, 4.

White K, Johansen AK, Nilsen M, Ciuclan L, Wallace E, Paton L, Campbell A, Morecroft I, Loughlin L, McClure JD, et al. Activity of the estrogen-metabolizing enzyme cytochrome P450 1B1 influences the development of pulmonary arterial hypertension. Circulation. 2012;126:1087-1098. doi: 10.1161/circulationaha.111.062927.

Table S1 List of Mouse primer sequences

Gene	Forward Primer	Reverse Primer
Gapdh	AGGTCGGTGTGAACGGATTTG	TGTAGACCATGTAGTTGAGGTCA
Notch3 (rat)	GCAGGTGATGGCCTAAGTTC	GGTCAGCCCCTACCCATTAT
Notch3	TGCCAGAGTTCAGTGGTGG	CACAGGCAAATCGGCCATC
Hes5	AGTCCCAAGGAGAAAAACCGA	GCTGTGTTTCAGGTAGCTGAC
HeyL	CAGCCCTTCGCAGATGCAA	CCAATCGTCGCAATTCAGAAAG
Hes1	ACACCGGACAAACCAAAGAC	AATGCCGGGAGCTATCTTTC
Hey1	CACCTGAAAATGCTGCACAC	ATGCTCAGATAACGGGCAAC
NOS3	CAGGACAACCTCATCCCTGT	CTGGCCTTCTGCTCATTTTC
SOD1	GAGACCTGGGCAATGTGACT	TTGTTTCTCATGGACCACCA
SOD2	GGCCAAGGGAGATGTTACAA	GCTTGATAGCCTCCAGCAAC
Nox4	CCAGAATGAGGATCCCAGAA	AGCAGCAGCAGCATGTAGAA
Trpm2	CTTTGGGGTGCAGTCAAGGAG	TCCATGAGCTAAGGTTTTCTTGC
RyR1	GAAGGTTCTGGACAAACACGGG	TCGCTCTTGTTGTAGAATTTGCGG
RyR2	GCAAGCCAGACTGCATGACC	AAATCGCAATGCCCAGCTTC
Cacna1s	CGTTCTCATCCTGCTCAACACC	GAGCTTCAGGATCATCTCCACTG
Cacna1g	GACCATGTGGTCCTCGTCATCA	TTTCAGCCAGGAAGACTGCCGT
Itpr3	AGCCAAGCAGACTAAACAGGAC	GCCGCTTGTTCACAGTTAAGTA
p115	TCCGGACCAAGAGTGGGGACAAGA	TCTCATCAGCCTCGACCTTT
LARG	CGTTGGTCTGGAAGGTGAAT	CACCGTGCTCAGCTTAATGA
PDZ	GAGTCTCGACCTTCCAGCAC	CTCTTGGGCTTCCCAATGTA
Bip	TTCAGCCAATTATCAGCAAACTCT	TTTTCTGATGTATCCTCTTCACCAGT
CHOP	CCACCACACCTGAAAGCAGAA	AGGTGAAAGGCAGGGACTCA
XBP1	CAGCACTCAGACTATGTGCA	GTCCATGGGAAGATGTTCTGG
ATF6	GGGTTCTGTCTTCCACTCCA	AAGCAGCAGAGTCAGGCTTTC
sGCα1	CCCCTGGTCAGGTTCCTAAG	GGAGACTCCCTTCTGCATTCT
sGCβ1	TGCTGGTGATCCGCAATTATG	GGTTGAGGACTTTGCTTGCAG
Rock1	GACTGGGGACAGTTTTGAGAC	GGGCATCCAATCCATCCAGC
Rock2	TTGGTTCGTCATAAGGCATCAC	TGTTGGCAAAGGCCATAATATCT

Primer sequences are in the 5’ to 3’ direction and were ordered from Eurofins Genomics (Ebersberg, Germany).

Table S2 List of antibodies used

Antibody target	Dilution	Supplier
anti-α-tubulin	1:10000	Abcam
anti-ß-actin	1:10000	Sigma-Aldrich
anti-Notch3	1:2000	Cell Signaling Technology
anti-Hes5	1:1000	Santa Cruz Biotechnology
anti-ET_AR	1:250	Santa Cruz Biotechnology
anti-ET_BR	1:250	Santa Cruz Biotechnology
anti-ET-1	1:500	Santa Cruz Biotechnology
anti-ROCK2	1:500	BD Biosciences
anti-ROCK1	1:500	Chemicon International
anti-phospho-eNOS (Thr_{495})	1:1000	Cell Signaling Technology
anti-phospho-eNOS (Ser_{1177})	1:1000	Cell Signaling Technology
anti-eNOS	1:1000	Santa Cruz Biotechnology
anti-BiP/GRP78	1:1000	Santa Cruz Biotechnology
anti-CHOP	1:500	Santa Cruz Biotechnology
anti-IRE1α	1:200	Santa Cruz Biotechnology
anti-phospho-IRE1α (Ser_{724})	1:500	Cell Signaling Technology
anti-Nox4	1:1000	Invitrogen
anti-phospho-MLC_{20} ($Ser_{18/19}$)	1:1000	Cell Signalling Technology
anti-SOD1	1:1000	Santa Cruz Biotechnology
anti-SOD2	1:1000	Cell Signaling Technology Cell
anti-PKG1	1:1000	Cell Signaling Technology
anti-sGC	1:500	Cayman Chemical
anti-α-SMA	1:200	Abcam

Table S3 Human patient cell line characteristics

Group	Sex	Age	Cell Line	Passage	Additional treatments/conditions
Control	Male	57	92mp	P4-6	Pneumonectomy
	Male	52	103mp	P3-6	Mild bronchiectasis, Adenocarcinoma
	Female	57	105mp	P4-7	COPD, Emphysema
	Male	61	110mp	P3-4	Adenocarcinoma
PAH	Female	30	73mp	P4-7	Heritable PAH (R899X), prostanoids, warfarin, frusemide
	Female	45	113mp	P4-7	Secondary PAH associated with congenital heart disease
	Female	52	117mp	P5	Secondary PAH associated with septal defect
	Male	61	120mp	P3-5	Idiopathic PAH on triple therapy (macitentan, sildenafil, Veletri)

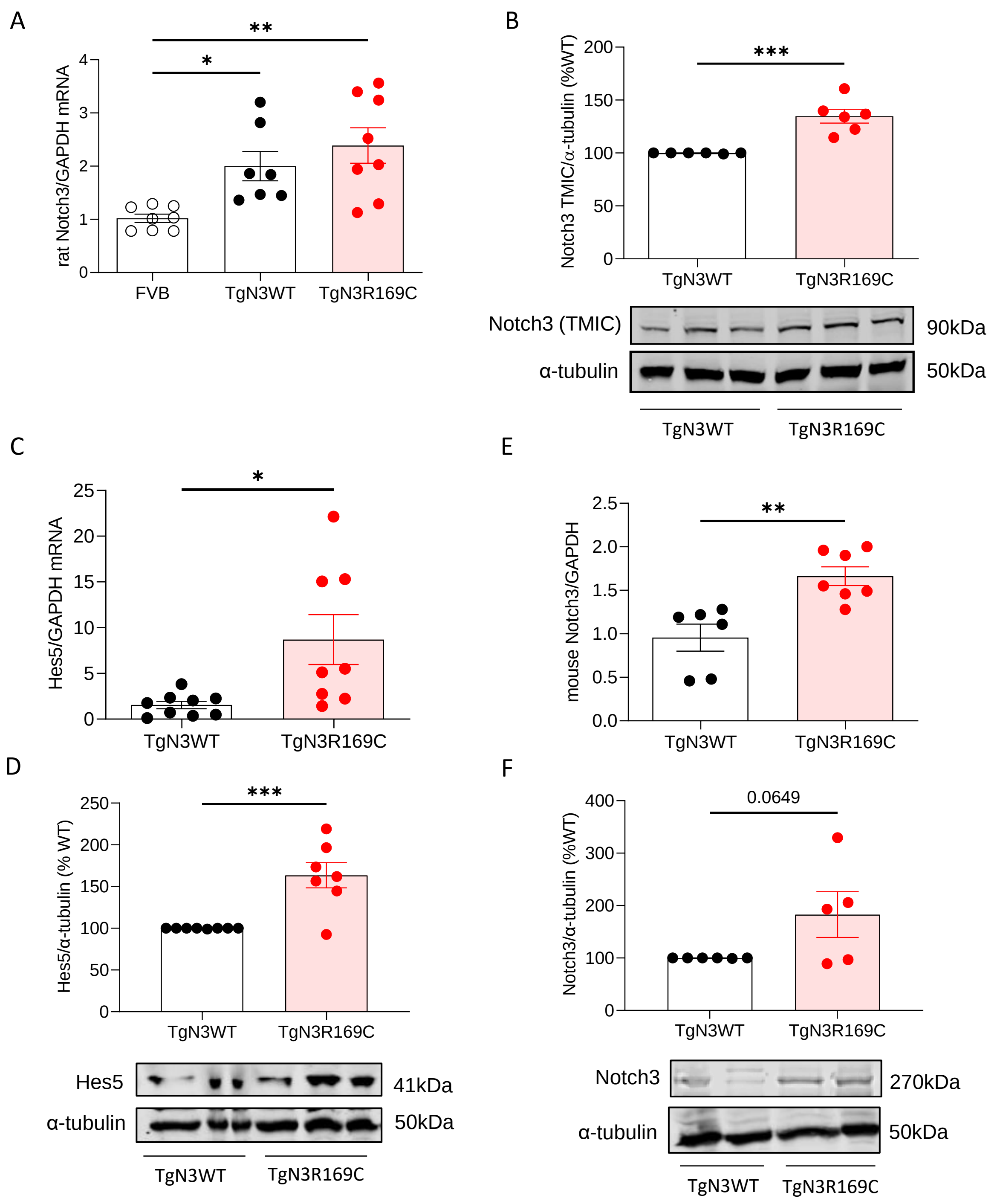

Figure S1. Notch3-Hes5 signalling is increased in lung of TgNotch3$_{R169C}$ mice. (A) TgNotch3$_{WT}$ and TgNotch3$_{R169C}$ mice overexpress the rat Notch3 transgene in whole lung when compared to non-transgenic wild type (FVB) littermates (n=7-8; one way ANOVA). (B) Upper panel: Quantification of levels of Notch3 transmembrane intracellular (TMIC) domain protein, a marker of Notch3 cleavage, in whole lung from TgNotch3$_{WT}$ and TgNotch3$_{R169C}$; Lower panel: Representative immunoblot of TMIC protein expression (n=6; unpaired Student's t-test). (C) Gene expression of Notch3 transcriptional target Hes5 in TgNotch3$_{WT}$ and TgNotch3$_{R169C}$ mouse lung. Analysis was by RT-qPCR with normalisation to GAPDH (n=8; unpaired Student's t-test). (D) Upper panel: Quantification of levels of Notch3 transcriptional target Hes5 protein in whole lung from TgNotch3$_{WT}$ and TgNotch3$_{R169C}$ normalised to α-tubulin; Lower panel: Representative immunoblot of Hes5 protein expression (n=8; unpaired Student's t-test). (E) Gene expression of Notch3 transcriptional target Notch3 in TgNotch3$_{WT}$ and TgNotch3$_{R169C}$ mouse lung. Analysis was by RT-qPCR with normalisation to GAPDH (n=8; unpaired Student's t-test). (F) Upper panel: Quantification of levels of mouse Notch3 protein in whole lung from TgNotch3$_{WT}$ and TgNotch3$_{R169C}$. Lower panel: Representative immunoblot of Notch3 protein expression in TgNotch3$_{WT}$ and TgNotch3$_{R169C}$ mouse lung normalised to α-tubulin. Results are expressed as mean±SEM. *p<0.05, ***p<0.001vs TgNotch3$_{WT.}$

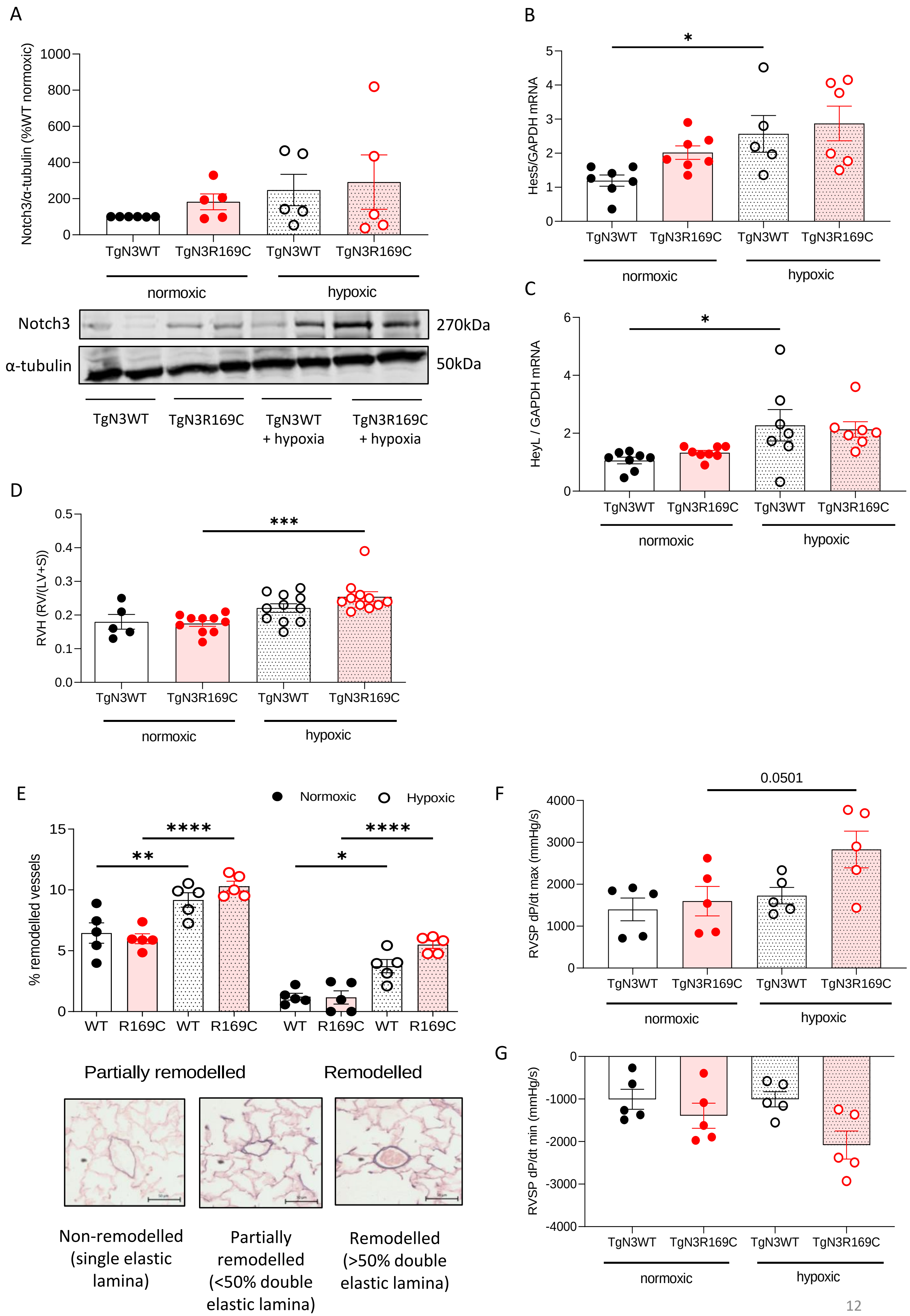
A
Notch3/α-tubulin (%WT normoxic)
1000
800
600
400
200
0
TgN3WT
TgN3R169C
TgN3WT
TgN3R169C
normoxic
hypoxic
Notch3
α-tubulin
270kDa
50kDa
TgN3WT
TgN3R169C
TgN3WT + hypoxia
TgN3R169C + hypoxia
B
Hes5/GAPDH mRNA
*
TgN3WT
TgN3R169C
TgN3WT
TgN3R169C
normoxic
hypoxic
C
HeyL / GAPDH mRNA
*
TgN3WT
TgN3R169C
TgN3WT
TgN3R169C
normoxic
hypoxic
D
RVH (RV/(LV+S))
***
TgN3WT
TgN3R169C
TgN3WT
TgN3R169C
normoxic
hypoxic
E
Normoxic
Hypoxic
% remodelled vessels
****
**
****
*
WT
R169C
WT
R169C
WT
R169C
WT
R169C
Partially remodelled
Remodelled
Non-remodelled (single elastic lamina)
Partially remodelled (<50% double elastic lamina)
Remodelled (>50% double elastic lamina)
F
0.0501
RVSP dP/dt max (mmHg/s)
TgN3WT
TgN3R169C
TgN3WT
TgN3R169C
normoxic
hypoxic
G
RVSP dP/dt min (mmHg/s)
TgN3WT
TgN3R169C
TgN3WT
TgN3R169C
normoxic
hypoxic

Figure S2. Chronic hypoxia further increases Notch3-Hes5 axis signalling and impairs vascular function in TgNotch3$_{WT}$. TgNotch3$_{WT}$ and TgNotch3$_{R169C}$ mice were exposed to chronic hypobaric hypoxia (10%) for 14 days. Notch3 signalling components were assessed at the gene level by RT-qPCR normalised to GAPDH, and at the protein level by immunoblot normalised to α-tubulin and expressed as percentage TgNotch$_{WT}$ normoxic group. (A) Expression of Notch3 protein in whole lung from normoxic and hypoxic TgNotch3$_{WT}$ and TgNotch3$_{R169C}$ (n=5; One-way ANOVA with Bonferroni post-test). Upper panel: Quantification of total Notch3 protein. Lower panel: Representative immunoblot of Notch3 protein. (B, C) Expression of mRNA for Notch3 targets Hes5 and HeyL (n=6-8; One-way ANOVA with Bonferroni post-test). (D) Right ventricular hypertrophy following hypoxia assessed by RV/(LV+S) (n=5-11; One-way ANOVA with Bonferroni post-test). (E) Upper panel: Quantification of remodelled vessels (% of total) with partial or full elastic lamina (n=5 animals per group, 107±26 vessels per animal; One-way ANOVA with Bonferroni post-test); Lower panel: Representative images of double elastic lamina remodelling in small pulmonary arteries assessed by elastin staining with picrosirius red counterstain RV contractility rate of contraction (G) and relaxation (H) was assessed by in vivo haemodynamics (n=5; One-way ANOVA with Bonferroni post-test). Data represent the mean±SEM. *p<0.05, **p<0.01, ***p<0.001.

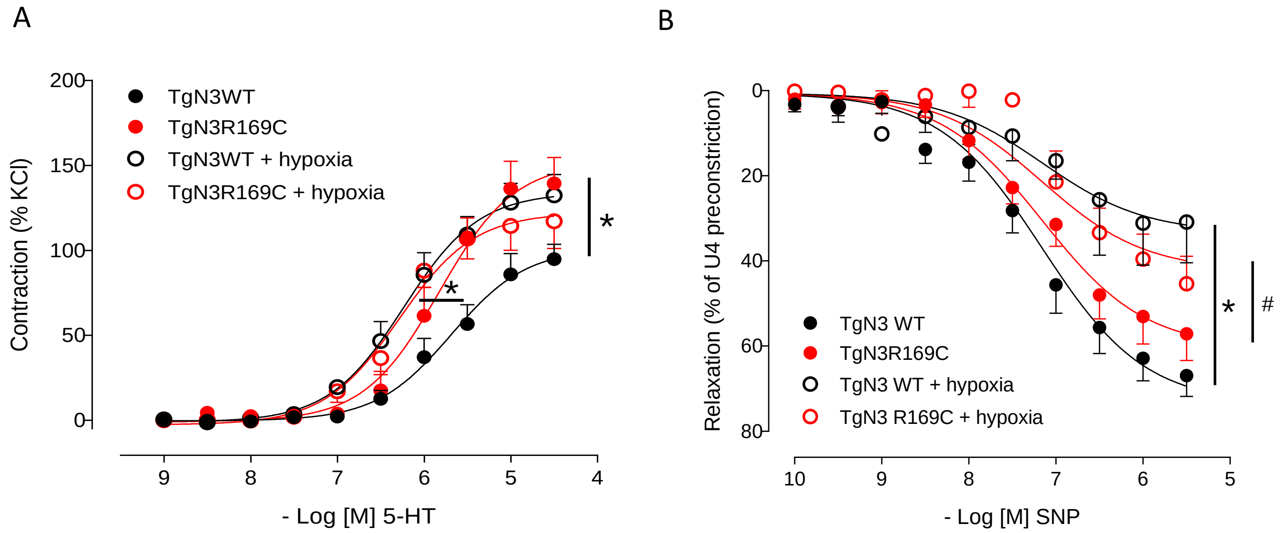

Figure S3 Additional vascular reactivity alterations in hypoxic vs normoxic TgNotch3 mice Vascular reactivity was assessed by wire myography in third order pulmonary arteries from hypoxic $TgNotch3_{WT}$ and $TgNotch3_{R169C}$ mice. Cumulative concentration-response curves of contractile responses to (B) 5-HT and relaxation response to (B) SNP were constructed in vessels from normoxic and hypoxic animals. Curves represent the mean±SEM for each group (n=4-10; non-linear regression fit). *p<0.05 vs $TgNotch3_{WT}$ normoxia, #p<0.05 vs $TgNotch3_{R169C}$ normoxia.

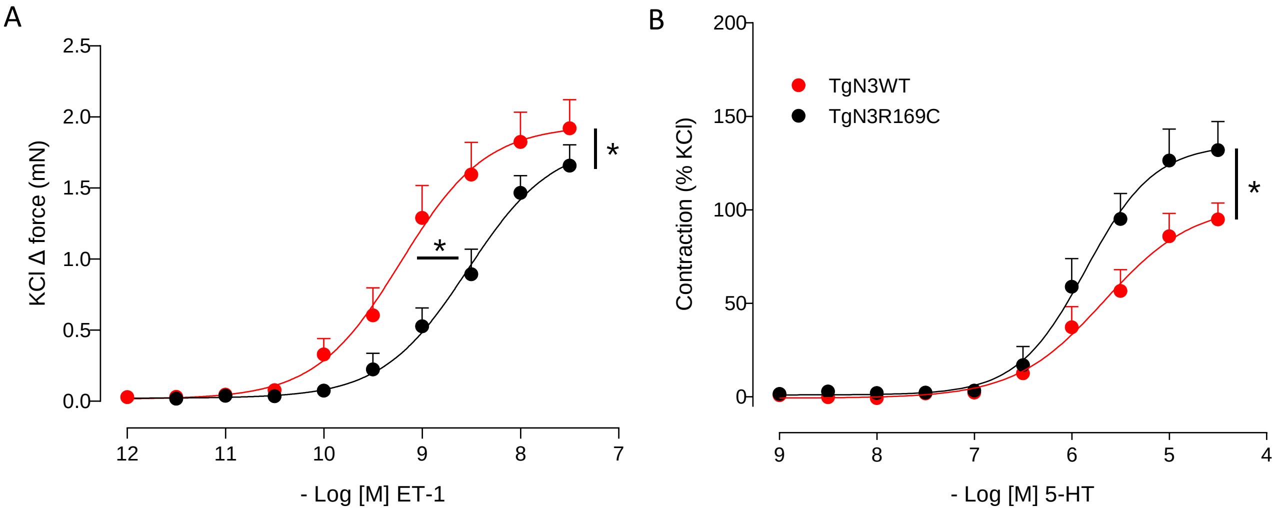

C

ET-1	Emax	logEC50 (M)
TgNotch3$_{WT}$	155.8 ± 10.87	-8.363 ± 0.1981
TgNotch3$_{R169C}$	190.8 ± 8.63%***	-9.152 ± 0.1419**
**5H-T**	Emax	logEC50 (M)
TgNotch3$_{WT}$	103.1 ± 12.79%	-5.764 ± 0.1826
TgNotch3$_{R169C}$	135.1 ± 11.03%**	-5.804 ± 0.1313

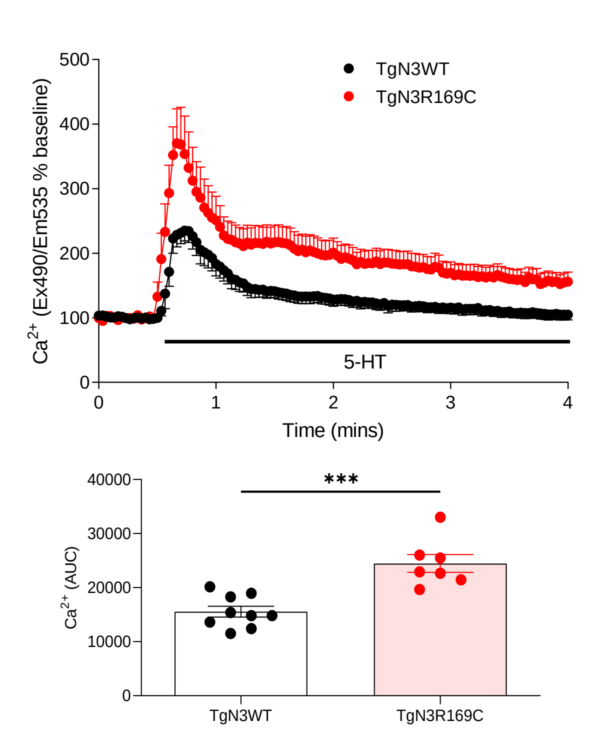

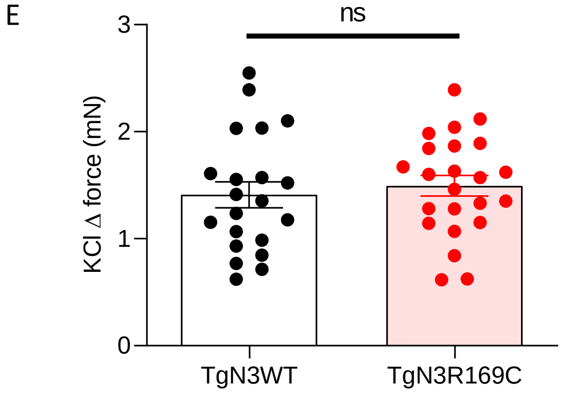

Figure S4. Further Ca^{2+}-dependent contractile data from normoxic TgNotch3 mice at baseline Vascular reactivity responses to ET-1 and 5-HT were assessed in third order pulmonary arteries from TgNotch3$_{WT}$ and TgNotch3$_{R169C}$ mice by wire myography. (A) ET-1 cumulative log-response curve presented as change in force (mN), not corrected for KCl (n=8-9; non-linear regression fit). (B) 5-HT cumulative log-response curve (n=7-9; non-linear regression fit). (C) Descriptive table of Emax and logEC$_{50}$ values for ET-1 and 5-HT mediated pulmonary artery contraction in TgNotch3 mice. (D) Intracellular Ca^{2+} transients to 5-HT were measured in TgNotch3$_{WT}$ and TgNotch3$_{R169C}$ PASMCs by live cell fluorescence. Upper panel: Representative tracings of PASMC $[Ca^{2+}]i$ responses to 5-HT (1µmol/L). Lower panel: $[Ca^{2+}]i$ calculated as the area under the curve (n=7-9; unpaired t-test). (E) KCl-induced pulmonary vascular contraction in TgNotch3 mice, expressed as mN of force (n=21-24; unpaired t-test). Results are expressed as mean±SEM. *$p<0.05$, **$p<0.01$, ***$p<0.001$ vs TgNotch3$_{WT}$.

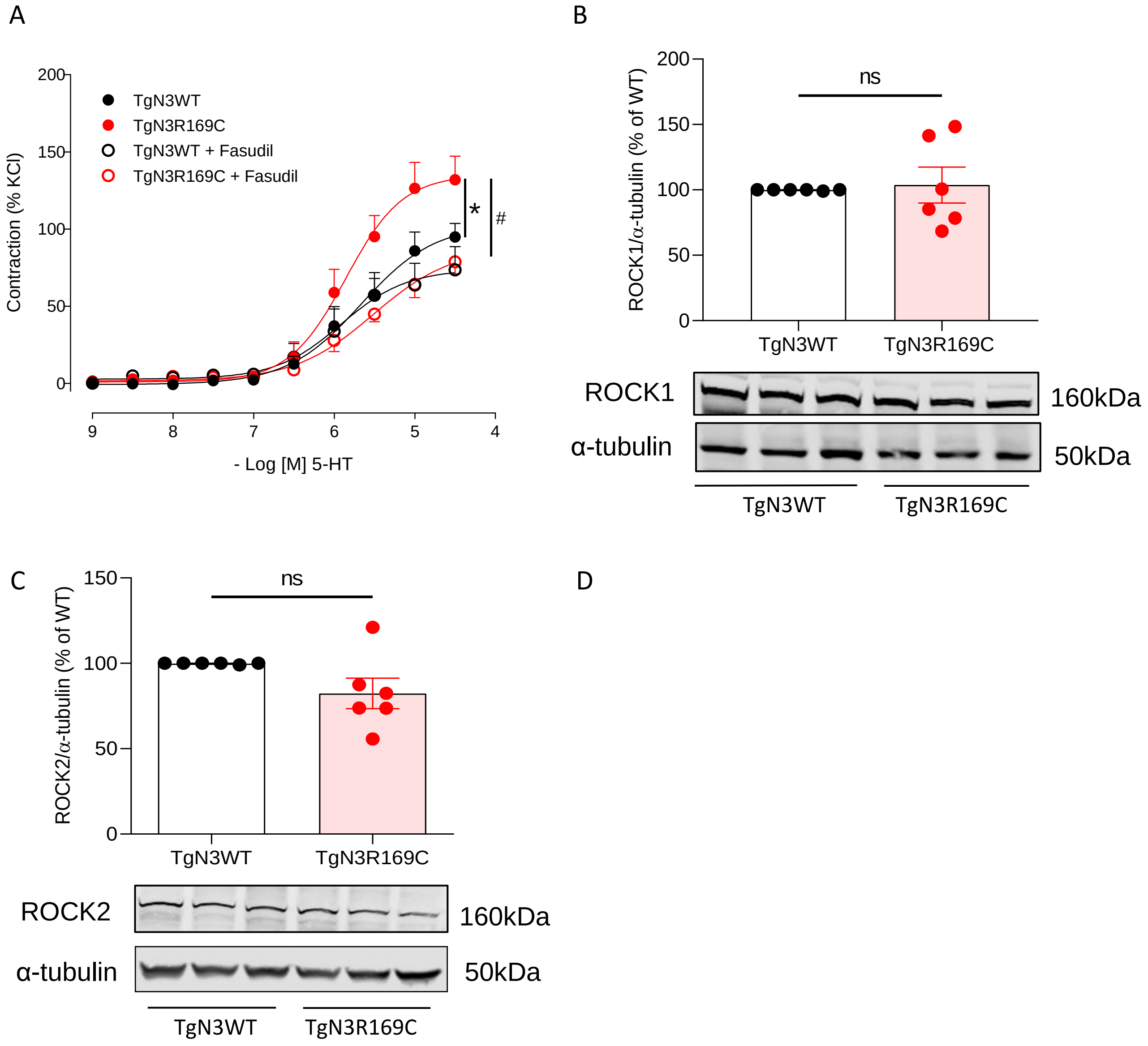

Figure S5. **Further ROCK in TgNotch3 lungs.** (A) Vascular reactivity responses to 5-HT were assessed in third order pulmonary arteries from TgNotch3$_{WT}$ and TgNotch3$_{R169C}$ mice by wire myography, +/- ROCK inhibitor fasudil (1µmol/L) (n=7-9; non-linear regression fit). ROCK expression was assessed immunoblot in whole lung from TgNotch3$_{WT}$ and TgNotch3$_{R169C}$ mice. (B) Upper panel: quantification of ROCK1 protein expression; Lower panel: representative immunoblot for ROCK1. (C) Upper panel: quantification of ROCK2 protein expression; Lower panel: Representative immunoblot for ROCK2 protein. (n=6; unpaired Student's t-test). Protein expression was normalised to α-tubulin. Results are expressed as mean±SEM. *$p<0.05$ vs TgNotch3$_{WT}$, #$p<0.05$ vs TgNotch3$_{R169C}$.

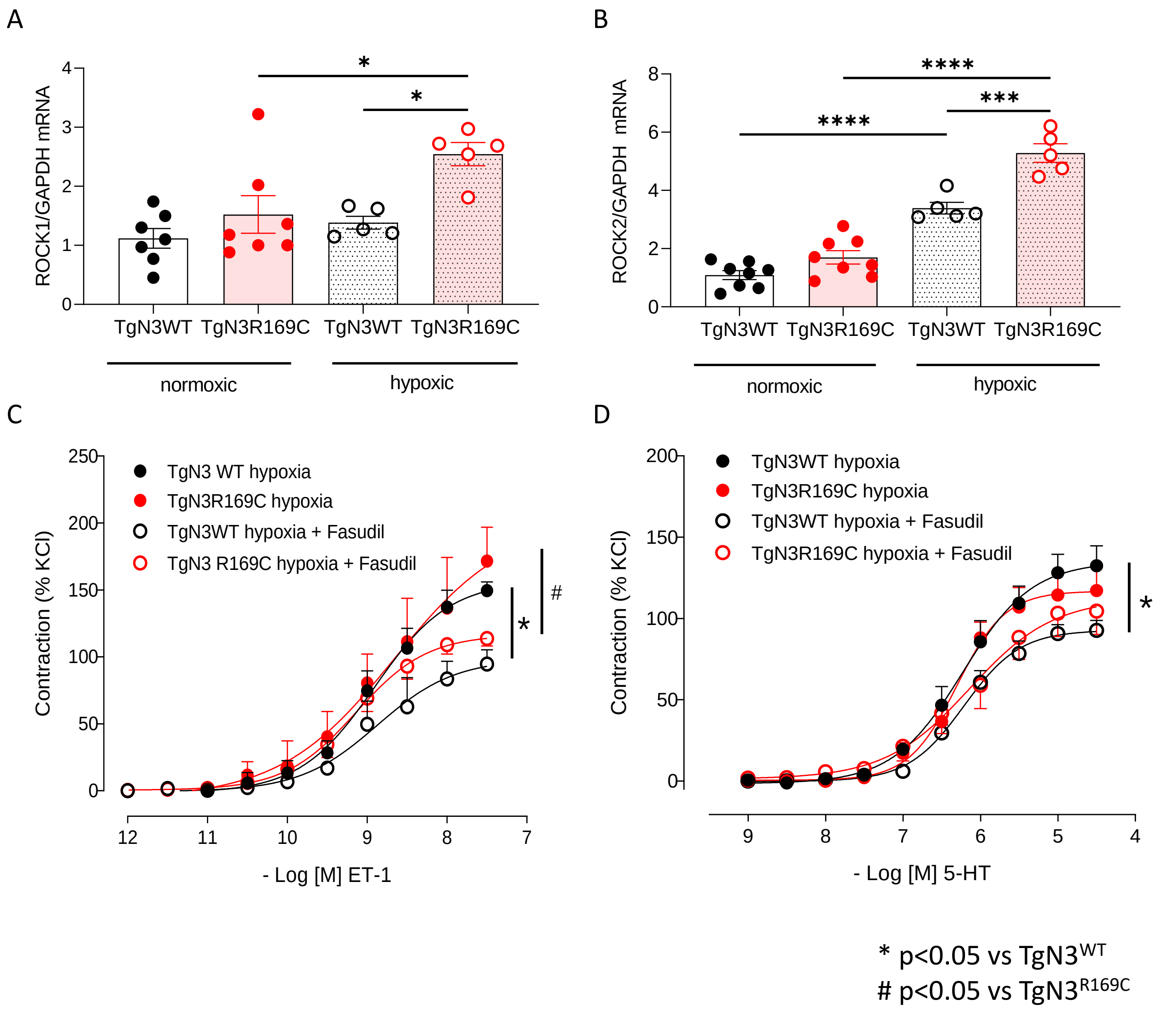

Figure S6. ROCK is implicated in vascular function in pulmonary arteries from chronic hypoxic TgNotch3$_{R169C}$ mice. Gene expression of ROCK was assessed by RT-qPCR, data expressed after normalisation to GAPDH and as fold change vs TgNotch3$_{WT}$ normoxic group. (A) ROCK1 mRNA expression, (B) ROCK2 expression (n=5-8; one-way ANOVA with Bonferroni post-test). Vascular reactivity was assessed by wire myography in third order pulmonary arteries from hypoxic TgNotch3$_{WT}$ and TgNotch3$_{R169C}$ mice. Cumulative concentration-response curves of contractile responses to (C) ET-1 and (D) 5-HT were constructed +/- Rho-kinase inhibitor fasudil (1µmol/L; 30 mins). Curves represent the mean±SEM for each group (n=3-9; non-linear regression fit). *p<0.05 vs TgNotch3$_{WT}$ hypoxia, #p<0.05 vs TgNotch3$_{R169C}$ hypoxia.

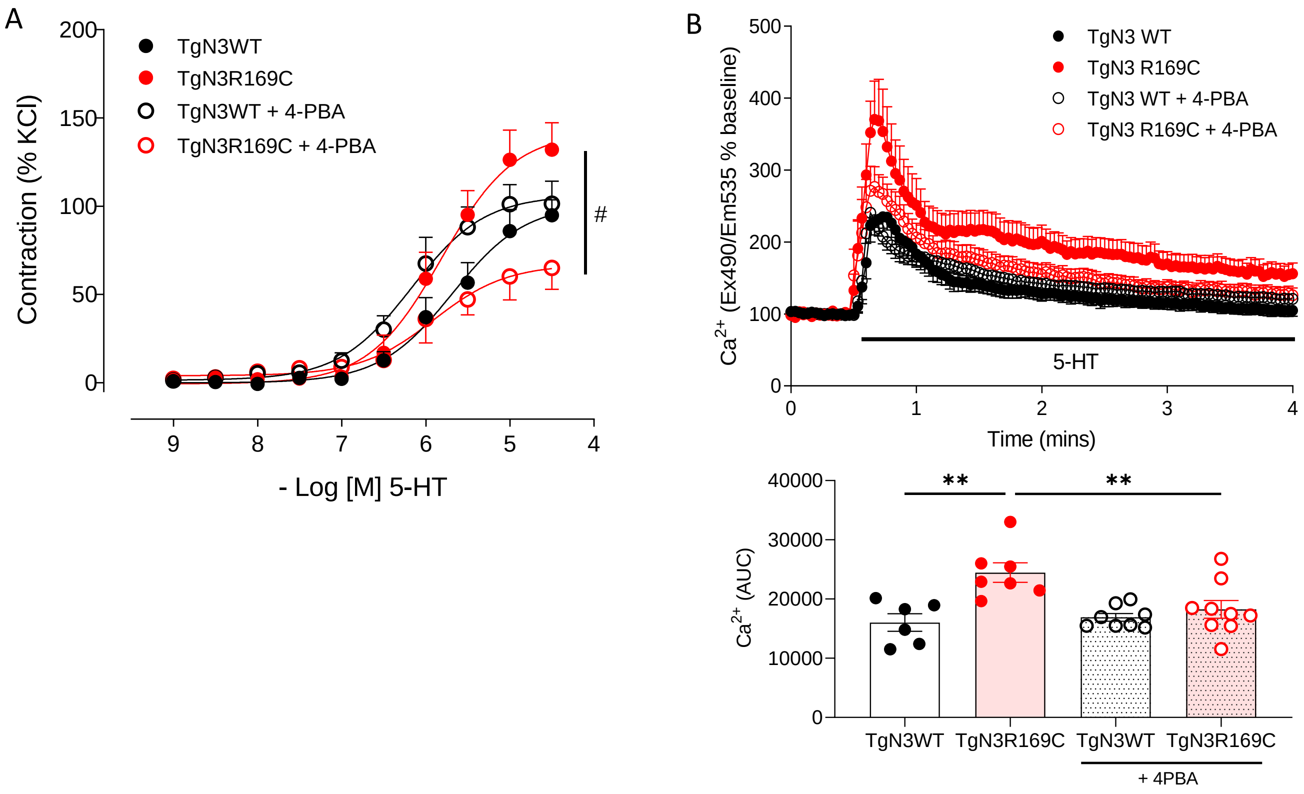

Figure S7. Further evidence of elevated ER stress data from TgNotch3$_{R169C}$ mice Vascular reactivity response to 5-HT was assessed in third order pulmonary arteries from TgNotch3$_{WT}$ and TgNotch3$_{R169C}$ mice by wire myography (n=5-9; non-linear regression fit). (A) 5-HT cumulative log-response curve +/- ER stress inhibitor 4-PBA (1mmol/L; 3 hrs). (B) Intracellular Ca^{2+} transients to 5-HT +/- ER stress inhibitor 4-PBA (1mmol/L; 3 hrs) were measured in TgNotch3$_{WT}$ and TgNotch3$_{R169C}$ PASMCs by live cell fluorescence. Upper panel: Representative tracings of PASMC $[Ca^{2+}]i$ responses to 5-HT (1μmol/L). Lower panel: $[Ca^{2+}]i$ calculated as the area under the curve (n=6-9; One-way ANOVA with Bonferroni post-test). Results are expressed as mean±SEM. *p<0.05, **p<0.01 vs TgNotch3$_{WT}$, #p<0.05 vs TgNotch3$_{R169C}$.

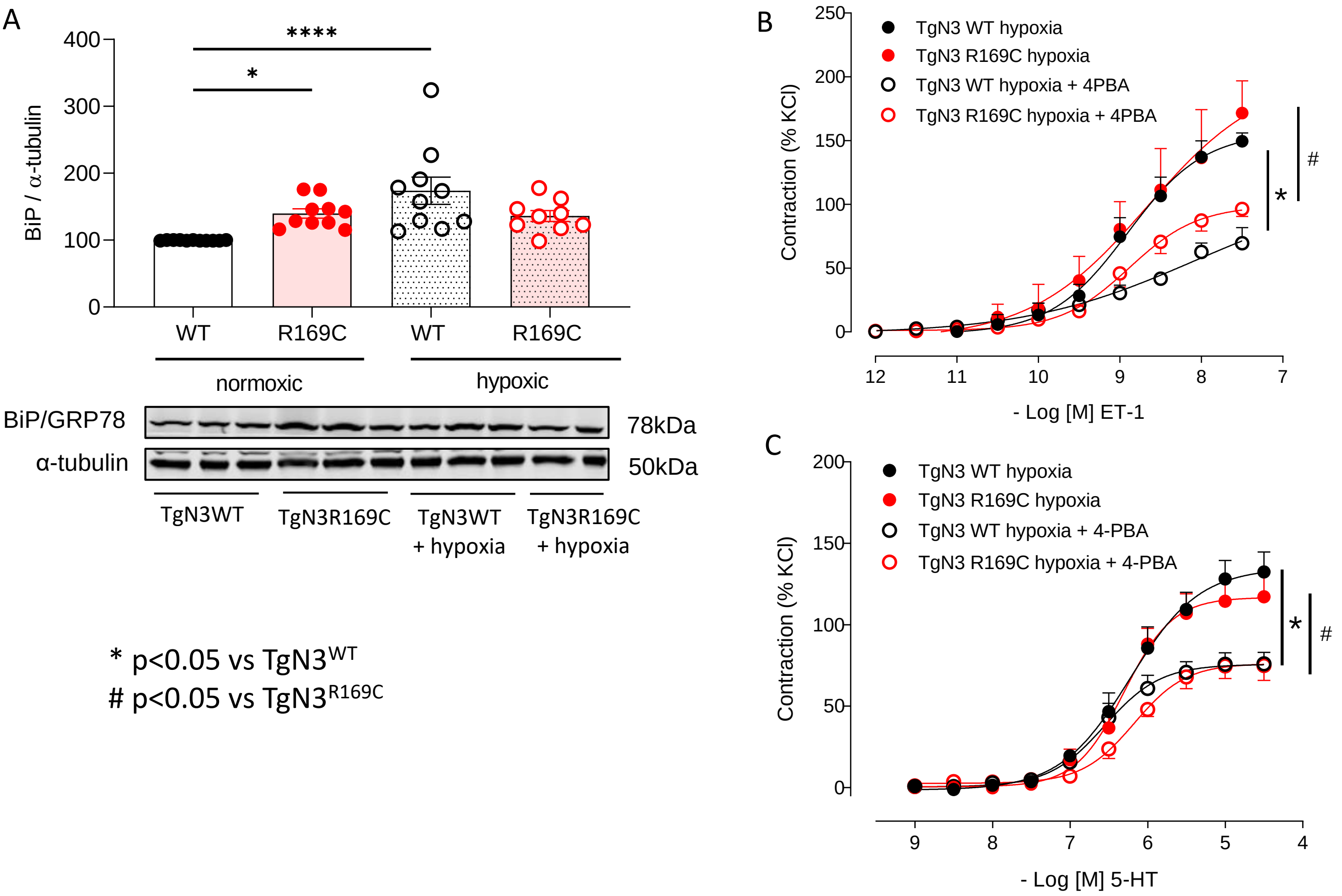

Figure S8. ER stress is implicated in vascular function in pulmonary arteries from chronic hypoxic TgNotch3$_{R169C}$ mice. Expression of ER stress marker and chaperone BiP/GRP78 was assessed by immunoblot with normalisation to α-tubulin, data expressed as percentage of TgNotch3WT normoxic group. Upper panel: Quantification of levels of BiP/GRP78 protein Lower panel: Representative immunoblot for BiP/GRP78 protein. Data presented as mean ± SEM. Vascular reactivity was assessed by wire myography in third order pulmonary arteries from hypoxic TgNotch3$_{WT}$ and TgNotch3$_{R169C}$ mice. Cumulative concentration-response curves of contractile responses to (B) ET-1 and (C) 5-HT were constructed +/- ER stress inhibitor 4-PBA (1mmol/L; 3 hrs). Curves represent the mean±SEM for each group (n=3-9; non-linear regression fit). *p<0.05 vs TgNotch3$_{WT}$, #p<0.05 vs TgNotch3$_{R169C}$.

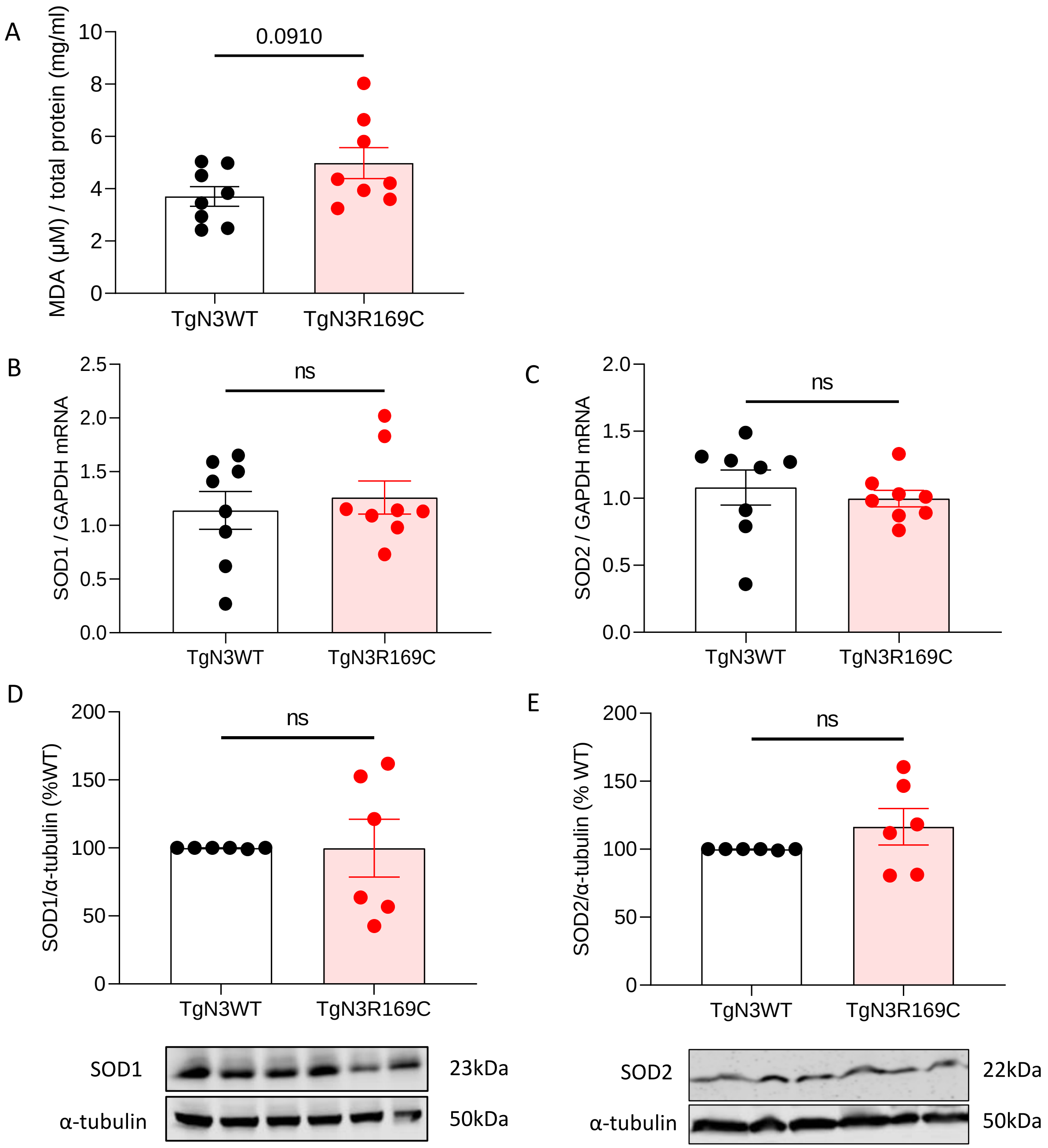

Figure S9. Altered ROS in TgNotch3$_{R169C}$ is not reliant on changes in superoxide dismutation (A) Lipid peroxidation was assessed in whole TgNotch3$_{WT}$ and TgNotch3$_{R169C}$ lung by TBARS assay for MDA levels, normalised to total protein (n=8; unpaired Student's t test). Gene expression for SOD1 (cytosolic) and SOD2 (mitochondrial) isoforms was assessed in whole lung from TgNotch3$_{WT}$ and TgNotch3$_{R169C}$ by RT-qPCR, with normalisation to GAPDH. (B) SOD1 mRNA expression, (C) SOD2 mRNA expression (n=8; unpaired Student's t-test). Protein expression was assessed by immunoblot with normalisation to α-tubulin, data is represented as percentage of TgNotch3$_{WT}$. (D) Upper panel: quantification of SOD1 protein expression; Lower panel: Representative immunoblot for SOD1 protein. (n=5; unpaired Student's t-test). (E) Upper panel: quantification of SOD2 protein expression; Lower panel: Representative immunoblot for SOD2 protein. (n=6; unpaired Student's t-test). Results are expressed as mean±SEM.

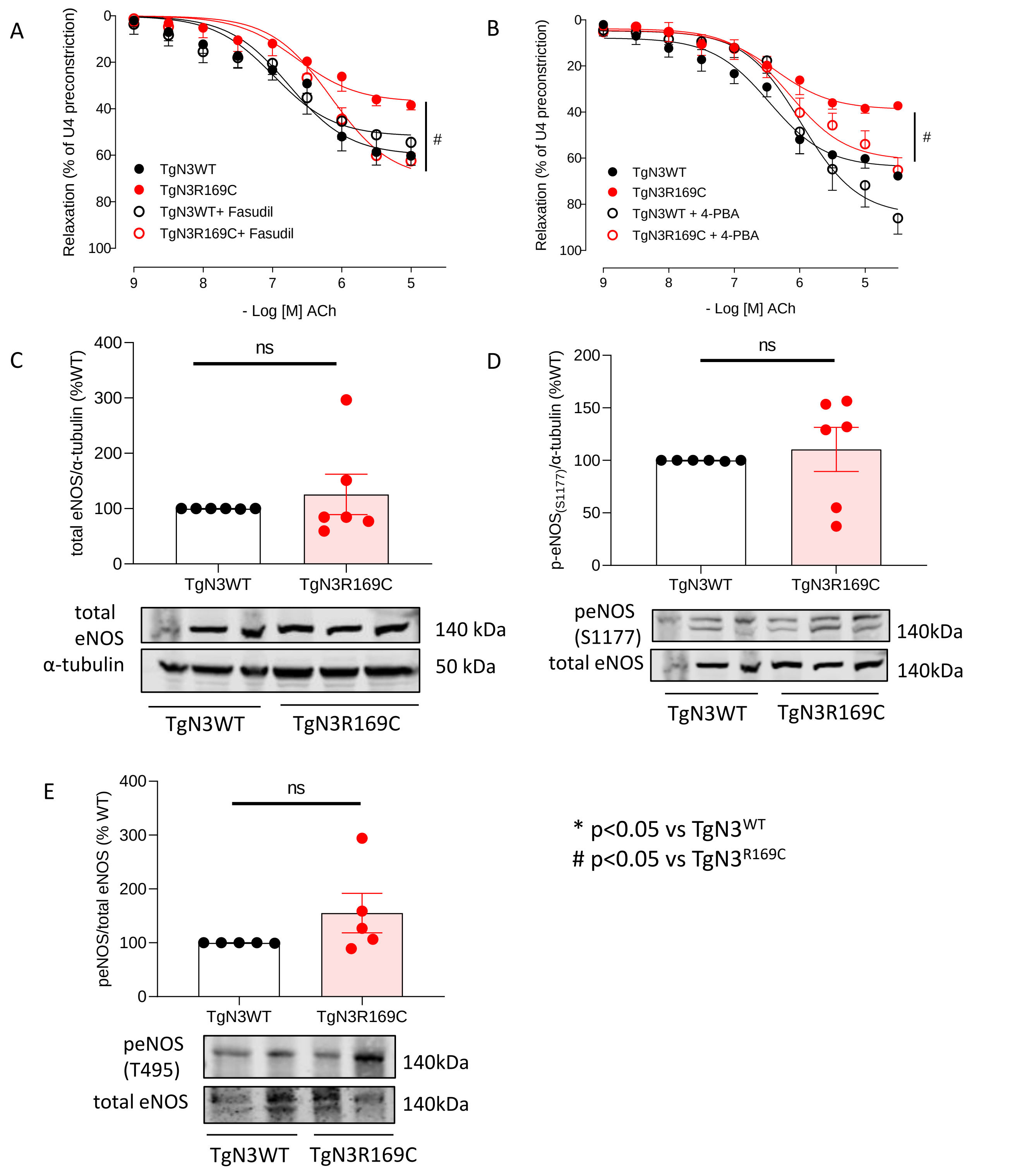

Figure S10. Reduced endothelial-dependent relaxation in TgNotch3$_{R169C}$ pulmonary arteries is not associated with alterations in eNOS expression or activation Vascular reactivity responses were assessed in third order pulmonary arteries from TgNotch3$_{WT}$ and TgNotch3$_{R169C}$ mice by wire myography. Cumulative concentration-response curves to ACh were contructed in TgNotch3 pulmonary arteries following +/- (A) ROCK inhibitor fasudil (1µmol/L; 30 mins), or (B) ER stress inhibitor 4-PBA (1mmol/L; 3 hrs) (n=5-9; non-linear regression fit). Protein expression was assessed by immunoblot with normalisation to α-tubulin/total eNOS, data is represented as percentage of TgNotch3$_{WT}$ (n=5-6 per group; unpaired Student's t-test). (C) Upper panel: quantification of total eNOS protein expression; Lower panel: Representative immunoblot for total eNOS protein. (D) Upper panel: quantification of eNOS phosphorylation at activation site Ser1177; Lower panel: Representative immunoblot for eNOS Ser1177 phosphorylation. (E) Upper panel: quantification of eNOS phosphorylation at inhibition site Thr495; Lower panel: Representative immunoblot for eNOS Thr495 phosphorylation. Data presented as mean ± SEM, *p<0.05 vs TgNotch3$_{WT}$, #p<0.05 vs TgNotch3$_{R169C}$.

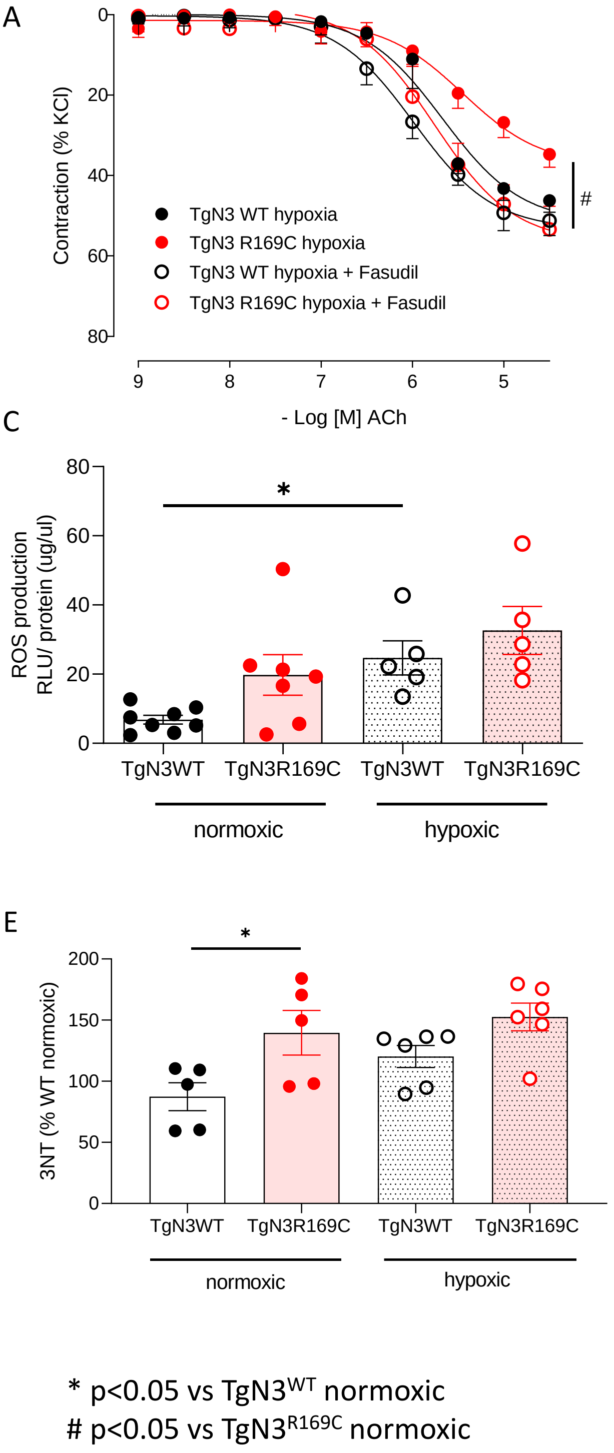

Figure S11. Chronic hypoxia further alters ROS in the lungs of TgNotch3 mice Vascular reactivity responses were assessed in third order pulmonary arteries from TgNotch3$_{WT}$ and TgNotch3$_{R169C}$ mice by wire myography. Cumulative concentration-response curves to ACh were constructed in hypoxic TgNotch3 mouse pulmonary arteries +/- (A) ROCK inhibitor fasudil (1µmol/L; 30 mins), or (B) ER stress inhibitor 4-PBA (1mmol/L; 3 hrs) (n=5-9; non-linear regression fit). (C) ROS levels in lung from normoxic and hypoxic TgNotch3 mice were assessed by chemiluminescence-enhanced lucigenin for O_2^- (n=5-8 per group, One-way ANOVA with Bonferroni post-test). (D), Amplex Red assay for H_2O_2 in lung from normoxic and hypoxic TgNotch3 mice (n=5 per group, One-way ANOVA with Bonferroni post-test). (E) 3-nitrotyrosine levels for $ONOO^-$ assay, and (F) nitrate levels for NO assay in normoxic and hypoxic TgNotch3 mouse lung (n=5-6 per group, One-way ANOVA with Bonferroni post-test). Data presented as mean ± SEM. *p<0.05, **p<0.01.

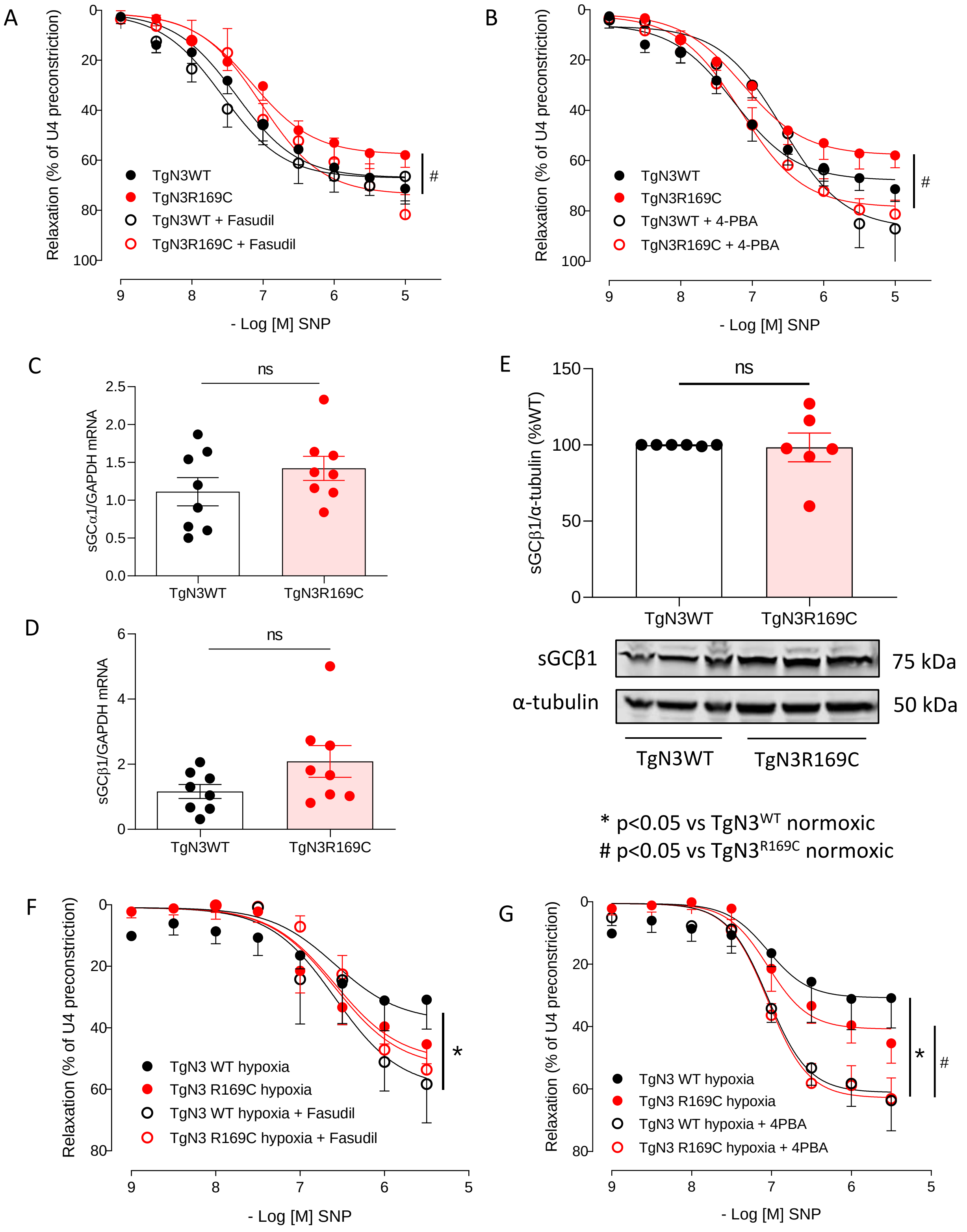

Figure S12. Reduced endothelial-independent relaxation in TgNotch3$_{R169C}$ pulmonary arteries is not associated with alterations in sGC expression Vascular reactivity responses were assessed in third order pulmonary arteries from TgNotch3$_{WT}$ and TgNotch3$_{R169C}$ mice by wire myography. Cumulative concentration-response curves to SNP were constructed in TgNotch3 pulmonary arteries +/- (A) ROCK inhibitor fasudil (1µmol/l; 30 mins), or (B) ER stress inhibitor 4-PBA (1mmol/L; 3 hours) (n=5-9; non-linear regression fit). Gene expression for sGC was assessed in TgNotch3 lung by RT-qPCR, with normalisation to GAPDH. (C) sGCα1 and (D) sGCβ1 mRNA expression (n=8 per group; unpaired Student's t-test). (E) sGCβ1 protein expression was assessed by immunoblot with normalisation to α-tubulin, data is represented as percentage of TgNotch3$_{WT}$ (n=6; unpaired Student's t-test). Upper panel: quantification of sGCβ1 protein expression; Lower panel: Representative immunoblot for sGCβ1 protein (pool of 3 animals per sample). (F, G) Cumulative concentration-response curves to SNP were also constructed in normoxic and hypoxic TgNotch3 pulmonary arteries +/- (A) ROCK inhibitor fasudil (1µmol/l; 30 mins), or (B) ER stress inhibitor 4-PBA (1mmol/L; 3 hours) (n=3-6; non-linear regression fit). Data presented as mean ± SEM, *$p<0.05$ vs TgNotch3$_{WT}$, #$p<0.05$ vs TgNotch3$_{R169C}$.

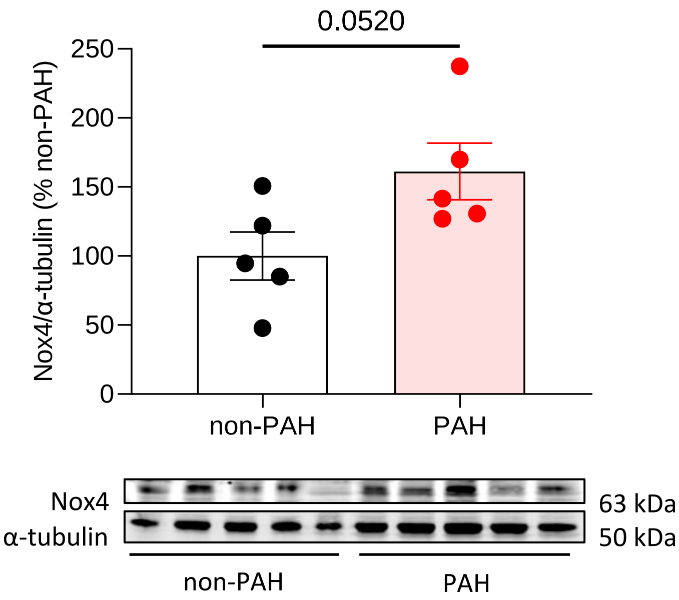

Figure S13. Nox4 expression in PAH vs non-PAH PASMCs Protein expression was assessed in PAH vs non-PAH PASMCs by immunoblot normalised to α-tubulin, data expressed as % of non-PAH. Upper panel: quantification of Nox4 expression; Lower panel: representative Nox4 immunoblot. (n=5, One-way ANOVA with Bonferroni post-correction). Results are expressed as mean±SEM.
