# Supplementary material for: Notch3/Hes5 Induces Vascular Dysfunction in Hypoxia-Induced Pulmonary Hypertension Through ER Stress and Redox-Sensitive Pathways
Source: Hypertension. 2023 May 31;80(8):1683–96. doi: 10.1161/HYPERTENSIONAHA.122.20449 (PMC10355806; doi:10.1161/HYPERTENSIONAHA.122.20449)
Supplement: Supplementary file 2 [file hyp-80-1683-s002.pdf]

*** Short In Vivo Checklist**

AHA - Preclinical Animal Testing : Prevention of bias is important for experimental cardiovascular research. ***This short checklist must be completed, and the answers should be clearly presented in the manuscript as well.*** *The checklist will be used by reviewers and editors but will not be published. If a revision is requested, you will be required to complete at revision submission a more detailed checklist that will be published with the accepted article.*

This study involves animals:
Yes

Animals

Species, age, sex, strains, and sources of animals are described: Yes

Randomization

Randomization and allocation concealment were performed: Yes

Blinding

Blinding was performed: Yes

Inclusions and Exclusions (a)

Specific criteria for inclusions and exclusions are specified: Yes

Inclusions and Exclusions (b)

Criteria for inclusions and exclusions were set before the study: Yes

Reporting of Excluded Animals

All animals excluded after the randomization are reported: Yes

Statistical Methods

Statistical Methods are described: Yes

Date completed: 04/19/2023 17:32:50
User pid: 770112
